# Coordinated Regulation of Virulence during Systemic Infection of *Salmonella enterica* Serovar Typhimurium

**DOI:** 10.1371/journal.ppat.1000306

**Published:** 2009-02-20

**Authors:** Hyunjin Yoon, Jason E. McDermott, Steffen Porwollik, Michael McClelland, Fred Heffron

**Affiliations:** 1 Department of Molecular Microbiology and Immunology, Oregon Health & Science University, Portland, Oregon, United States of America; 2 Pacific Northwest National Laboratories, Richland, Washington, United States of America; 3 The Sydney Kimmel Cancer Center, San Diego, California, United States of America; Yale University School of Medicine, United States of America

## Abstract

To cause a systemic infection, *Salmonella* must respond to many environmental cues during mouse infection and express specific subsets of genes in a temporal and spatial manner, but the regulatory pathways are poorly established. To unravel how micro-environmental signals are processed and integrated into coordinated action, we constructed in-frame non-polar deletions of 83 regulators inferred to play a role in *Salmonella enteriditis* Typhimurium (STM) virulence and tested them in three virulence assays (intraperitoneal [i.p.], and intragastric [i.g.] infection in BALB/c mice, and persistence in 129X1/SvJ mice). Overall, 35 regulators were identified whose absence attenuated virulence in at least one assay, and of those, 14 regulators were required for systemic mouse infection, the most stringent virulence assay. As a first step towards understanding the interplay between a pathogen and its host from a systems biology standpoint, we focused on these 14 genes. Transcriptional profiles were obtained for deletions of each of these 14 regulators grown under four different environmental conditions. These results, as well as publicly available transcriptional profiles, were analyzed using both network inference and cluster analysis algorithms. The analysis predicts a regulatory network in which all 14 regulators control the same set of genes necessary for *Salmonella* to cause systemic infection. We tested the regulatory model by expressing a subset of the regulators *in trans* and monitoring transcription of 7 known virulence factors located within *Salmonella* pathogenicity island 2 (SPI-2). These experiments validated the regulatory model and showed that the response regulator SsrB and the MarR type regulator, SlyA, are the terminal regulators in a cascade that integrates multiple signals. Furthermore, experiments to demonstrate epistatic relationships showed that SsrB can replace SlyA and, in some cases, SlyA can replace SsrB for expression of SPI-2 encoded virulence factors.

## Author Summary

We have used the intracellular pathogen *Salmonella* to investigate how pathogenic bacterium correctly expresses virulence factors during infection. After ingestion by its host, *Salmonella* confronts multiple challenging microenvironments such as acidic pH in the stomach, nutrient starvation, and host innate immune responses. However, *Salmonella* overcomes host defenses through expression of an array of virulence factors. We defined 14 *Salmonella* regulators out of 83 tested that are required for systemic infection. We then analyzed expression of *Salmonella* genes in each mutant strain grown under various conditions. Surprisingly, these regulators coordinately activate expression of a subset of virulence genes required for intracellular survival and replication. Computer-aided analysis based on our data established a regulatory network in which all 14 regulators are connected. Furthermore, the analysis identified many additional genes with no known function that are expressed under conditions identical to those that express known virulence factors. This work suggests that many environmental signals are processed through diverse regulators and integrated to induce virulence genes allowing *Salmonella* to survive and multiply within its host.

## Introduction

Gastrointestinal infections are the second most common cause of childhood mortality in the developing world and Typhoid alone (caused by serovar Typhi) is estimated to result in 500,000 deaths per year [1]. In addition to fluid and electrolyte loss, non-typhoidal *Salmonella* often results in septicemia in children and in HIV infected adults in developing countries with a fatality rate of 25% or greater [2]. *Salmonella enteriditis* serotype Typhimurium (referred to simply as *Salmonella* or Typhimurium below) is a paradigm for understanding intracellular pathogenesis because of its established genetics and simple and inexpensive animal model - the mouse. All strains of *Salmonella enteriditis* share at least 95% sequence identity; the differences are associated with growth in a specific host or survival in an environmental niche. More than 4% of the entire genome is required for Typhimurium to infect the mouse [3]. These genes are widely distributed around the entire circular chromosome including many genes not involved in metabolic processes nor required for growth under laboratory conditions. Numerous studies have assigned a small fraction of these genes to specific steps in mouse infection but most are still a mystery. Many virulence genes are attributable to horizontally acquired DNA sequences that are not present in nonpathogenic but related bacteria. These regions include two 40 kb stretches of DNA termed *Salmonella* pathogenicity islands 1 (SPI-1) and 2 (SPI-2) [4]–[9]. SPI-1 and SPI-2 encode a secretion apparatus resembling a needle and related to the bacterial flagella that uses the proton motive force to secrete proteins directly into the cytoplasm of the eukaryotic cell [10]. Secretion can take place from extracellular bacteria that are juxtaposed to the surface of the cell or from intracellular bacteria located in vacuoles. The two type III secretion systems are expressed under different environmental conditions and play distinct roles in pathogenesis. SPI-2 is known to be required for systemic infection whereas SPI-1 plays an essential role during intestinal infection and in mouse persistence [11]–[14].

During the course of systemic infection in mice, bacteria are found within neutrophils, monocytes, dendritic cells, and B and T cells but are not found extracellularly until the last one or two days immediately before death of the mouse [15]–[17]. How *Salmonella* survives and replicates within the host and how it expresses virulence genes at the appropriate time during systemic infection is little understood and the subject of this work. Technological advances in the last 10 years such as microarrays, whole genome sequences, and global proteomics have provided a more complete picture of gene expression for a number of bacteria. The goal of the current work is to develop a predictive model for host-pathogen interactions that will provide insight into how *Salmonella* responds to specific conditions in the host. This approach was based on identification of regulators that were necessary for *Salmonella* to cause a systemic infection and transcriptional profiling of isogenic derivatives missing the regulator under a variety of growth conditions. The transcriptional profiles provided more than 300,000 data point, necessitating computer analysis. We have used SEBINI (Software Environment for Biological Network Inference; [18]) to directly compare multiple network algorithms. The network inference algorithm that we have used is the context likelihood of relatedness (CLR) to analyze the gene expression profiles [19]. CLR is an extension of the relevance network class of machine learning algorithms [20] and provides the highest precision of several algorithms tested [19]. At a 60% true positive rate, CLR identified 1,079 regulatory interactions in *E. coli*, of which 338 were in previously known networks and 741 were novel predictions (ibid). The analysis of our data provided a testable regulatory hierarchy and a list of genes with similar expression profiles as described below.

## Results

### *Salmonella* regulators required for systemic mouse infection

**Figure 1:**
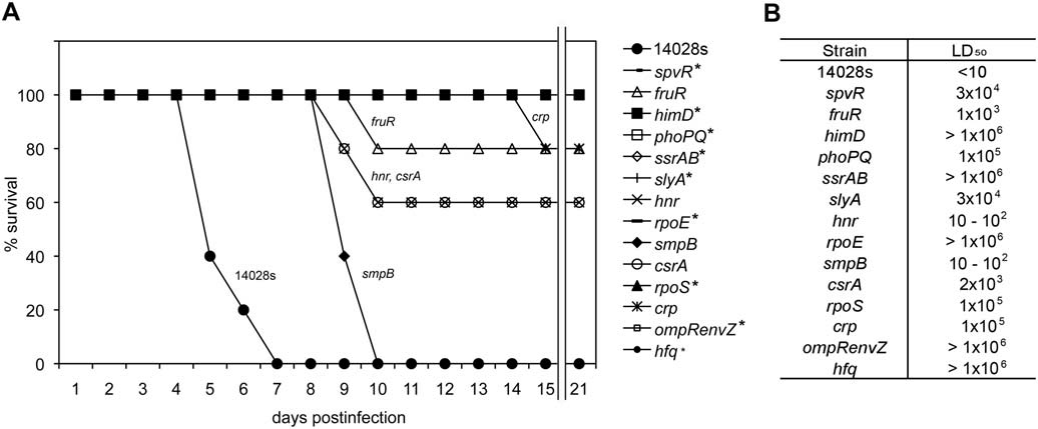
Attenuation of *S. typhimurium* strains encoding deletions of the indicated regulatory gene. A. Five BALB/c mice were i.p. infected with ∼200 cfu (approximately 100× LD_50_) of the *Salmonella* strain indicated and observed for 21 days. The percentages of surviving mice are shown for each strain. *Salmonella* strains lacking *smpB*, *hnr*, *csrA*, *fruR*, or *crp* caused death of some mice during the observation period and the other strains indicated with an asterisk resulted in no deaths. B. Each *Salmonella* strain was administered i.p. to 3 groups of 3 BALB/c mice at 1×10^2^, 1×10^4^, and 1×10^6^ cfu respectively and monitored for 1 month to estimate the approximate LD_50_ values.

Typhimurium encodes a surfeit of regulators (more than 330 based on annotation cited in NCBI) presumably because it can survive and replicate in many different environments, cause infection in diverse hosts, and can use multiple carbon sources and terminal electron acceptors. We focused on 83 regulators presumed to play roles in virulence, based on published data including negative selection experiments [13], [21]–[23]. We constructed non-polar in-frame deletions in which each regulator gene was replaced with a “scar” sequence using bacteriophage lambda-mediated recombination [24],[25]. The list of 83 regulator genes is provided in Supporting Information (Table S1). For the two-component regulator *ssrA*/*ssrB* we constructed in-frame deletions missing *ssrA*, *ssrB* or both; for the other two two-component regulators, *phoP*/*phoQ* and *ompR*/*envZ*, both the signal sensor and response regulator were deleted. As an initial screen two 4–6 weeks-old BALB/c mice were infected intraperitoneally (i.p.) with 200 colony forming units (CFU) of each deleted strain (about 100× the LD_50_). Mutations that resulted in either no deaths, one death, or delayed death of infected mice were retested with groups of 5 mice at the same dose (Figure 1A). Based on this preliminary screen we chose the regulators *spvR*, *fruR*, *himD*, *phoP*/*phoQ*, *ssrA*/*ssrB*, *slyA*, *hnr*, *rpoE*, *smpB*, *csrA*, *rpoS*, *crp*, *ompR*/*envZ*, and *hfq* for further investigation (see Figure 1 and Table 1 for references and descriptions; a complete list of virulence phenotypes is provided in Table S1). It is possible that additional regulators would be identified if a larger group of mice were used in the initial screen. The LD_50_ for each of the mutants was computed as shown in Figure 1B. Compared to the parental strain (ATCC14028), which has an LD_50_ of 1–2 cfu, all of the derivatives were attenuated for virulence. The 14 deletion strains lacking regulators reported in this study were selected from i.p. BALB/c mice infection to eliminate other regulators required only for gastrointestinal infection or persistence but most of these strains were avirulent in another strain of mice (129X1/SvJ) and by other route (intragastric) of infection (see Table S1). Therefore the regulators investigated in this study might be considered the most central regulators for virulence. In the other virulence assays, the 83 derivatives were tested for virulence by intragastric (i.g.) BALB/c infection and by a more sensitive competitive index (CI) experiment. In the competitive index experiment all mutants were co-administered to 129X1/SvJ mice and the number of each surviving mutant bacteria was determined 7 days after i.p. inoculation. BALB/c mice are missing natural resistance-associated macrophage protein (Nramp1) and thus succumb to Typhimurium infection within a week when mice are infected i.p. with less than 10 bacteria of the strain employed here (14028). Unlike BALB/c, 129X1/SvJ mice have a functional copy of Nramp1 and the bacteria persist for several weeks without killing the mouse [13],[26]. The CI test identified 30 (out of 83) regulator mutants that were attenuated in comparison to the wild type control (see Table S1). The 14 derivatives chosen for this study were among those showing the poorest survival by comparison to the parent in this competitive index experiment.

**Table 1:** List of virulence regulators analyzed in this study, gene number, gene symbol, description and reference.

Gene no.	Gene symbol	Description	Reference
pSLT041	*spvR*	*Salmonella* plasmid virulence: regulation of *spv* operon (LysR family)	[42]
STM0118	*fruR*	transcriptional repressor of *fru* operon and others (GlaR/LacI family)	[95]
STM0982	*himD*	integration host factor (IHF), beta subunit; site-specific recombination	[32]
STM1230/1	*phoQ*/*phoP*	two-component regulatory system responding to Mg^+^	[28]
STM1391/2	*ssrB*/*ssrA*	two-component regulatory system	[7],[8]
STM1444	*slyA*	transcriptional regulator for hemolysin (MarR family)	[31]
STM1753	*hnr*	response regulator in protein turnover	[96]
STM2640	*rpoE*	sigma E factor of RNA polymerase, response to periplasmic stress	[27]
STM2688	*smpB*	small protein B; tmRNA-binding protein	[97]
STM2826	*csrA*	carbon storage regulator	[30]
STM2924	*rpoS*	sigma S factor of RNA polymerase, major sigma factor during stationary phase	[98]
STM3466	*crp*	catabolite activator protein (CAP), cyclic AMP receptor protein (CRP family)	[99]
STM3501/2	*envZ*/*ompR*	two-component regulatory system responding to osmolarity and pH	[100]
STM4361	*hfq*	host factor I for bacteriophage Q beta replication, a growth-related protein	[33]

The 14 regulators identified were diverse and included alternative sigma factors (*rpoS* and *rpoE*), two-component regulators (*ompR/envZ*, *phoP/phoQ*, and *ssrA/ssrB*), a response regulator for which the signal sensor is unknown (*hnr* or *mviA*), post-transcriptional regulators (*csrA*, *hfq* and *smpB*), a bending protein essential for some types of recombination (*ihf*) and an assortment of other DNA binding proteins (*fruR*, *spvR*, *crp*, and *slyA*). Transcription profiling was carried out for isogenic deletion mutants in each of these 14 genes under four different conditions and analyzed to infer a regulatory hierarchy during systemic infection. *Salmonella* transcription profiles for the following regulators have already been reported: *rpoE* [27]; *phoP*/*phoQ* [28]; *ssrA*/*ssrB* [29]; *csrA* [30]; *slyA* [31]; *ihf* [32]; *hfq* [33]. Our results closely match these published results.

The large degree of attenuation we observed for an *spvR* mutation following i.p. infection was surprising, as most studies had limited its effect to the i.g. route of infection. To validate this result we complemented the *spvR* mutation *in trans* and found complete complementation in mouse virulence (Figure S1). The differences between our results and others are most likely related to the different strains of *Salmonella* used in the studies [34]. For the other mutations we have mobilized the mutation into a new genetic background by P22 transduction to ensure that there is no secondary mutation that influences the results [35].

### The ability of *Salmonella* to survive and replicate in macrophages is necessary but not sufficient for mouse virulence

**Figure 2:**
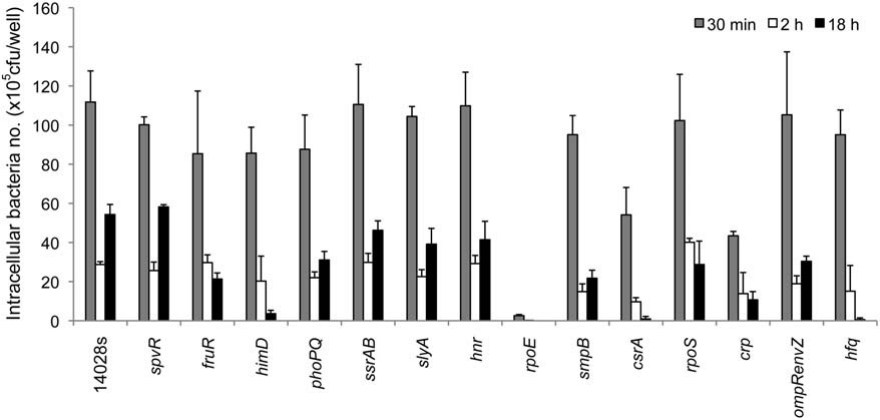
Survival of *Salmonella* strains containing mutations in each of 14 virulence regulators in primary macrophages. Primary bone marrow-derived macrophages were prepared as described in Materials and Methods and infected at an input MOI of 100 by low speed centrifugation onto cells in identically seeded wells (on average our conditions result in 1–2 intracellular bacteria per cell). The bacteria were opsonized in 10% normal mouse serum for 20 min prior to infection. Intracellular survival was measured at 30 min (grey), 2 h (white) and 18 h (black) post infection from at least three independent assays. Times refer to the time after centrifugation for macrophage infection. The results suggest that there is not a one to one correlation between systemic mouse infection and survival/replication in primary macrophages.

Previous work demonstrated that *Salmonella* mutants that were unable to survive within elicited peritoneal macrophages were attenuated for virulence during systemic mouse infection [36]. In fact, fluorescence-activated cell sorting analysis of infected blood and spleen using *Salmonella* that expresses green fluorescent protein does not identify any extracellular bacteria [16],[37],[38]. *Salmonella* is within blood monocytes and in other WBC in the spleen including neutrophils, dendritic cells, and B and T cells in these reports (ibid). It is possible that growth in cells types other than macrophages is necessary for *Salmonella* to cause a systemic infection in mice following i.p. administration. Thus, some of the regulatory mutations described here may affect growth in cells types other than macrophages. We therefore wished to determine if there is a direct relationship between growth in macrophages and mouse virulence. In these studies we used primary bone marrow-derived macrophages (BMDM) from the same strain of mouse as used in the original identification of attenuated regulatory mutants (BALB/c). The identical number of input bacteria and the identical number of macrophages were used in every infection experiment. As observed by others, following phagocytosis there is some bacterial killing that varied from strain to strain followed by intracellular growth. We monitored the number of intracellular bacteria at an early time (30 min) to determine the number of bacteria internalized (Figure 2). Even at the shortest time few intracellular bacteria were recovered from macrophage infection with an *rpoE* mutant, suggesting that this strain is very sensitive to microbicidal factors released by macrophages on contact with bacteria or doesn't get internalized very well. At 2.0 hrs post infection there was a decrease in bacterial numbers that varied from strain to strain presumably reflecting variation in the sensitivity to bacterial killing by the oxidative burst, acidic pH, and antimicrobial peptides. Finally, at 18 hrs bacterial numbers were enumerated to monitor intracellular replication as well as the ability to withstand nitrous oxide oxidation and other late antimicrobial factors [39],[40]. No effect was found at any time point for a mutant in the plasmid-encoded regulator *spvR* in murine macrophages in agreement with other investigators [41],[42]. Small differences in intracellular growth were observed for *ssrA*/*ssrB* and *slyA* compared to the parent although larger differences have been observed previously [43]–[45] perhaps reflecting BMDM preparation techniques, bacterial strain differences, or opsonization differences [46]. Mutations of *himD*, *rpoE*, *crp*, or *hfq* drastically reduce the number of viable bacteria that can be recovered from macrophages even at short times (Figure 2). These results also demonstrated that growth in macrophages *per se* does not duplicate *in vivo* infection and that some regulator mutants that were totally avirulent in the mouse showed no differences in growth in these primary macrophages.

### Transcription profiling of avirulent strains

**Figure 3:**
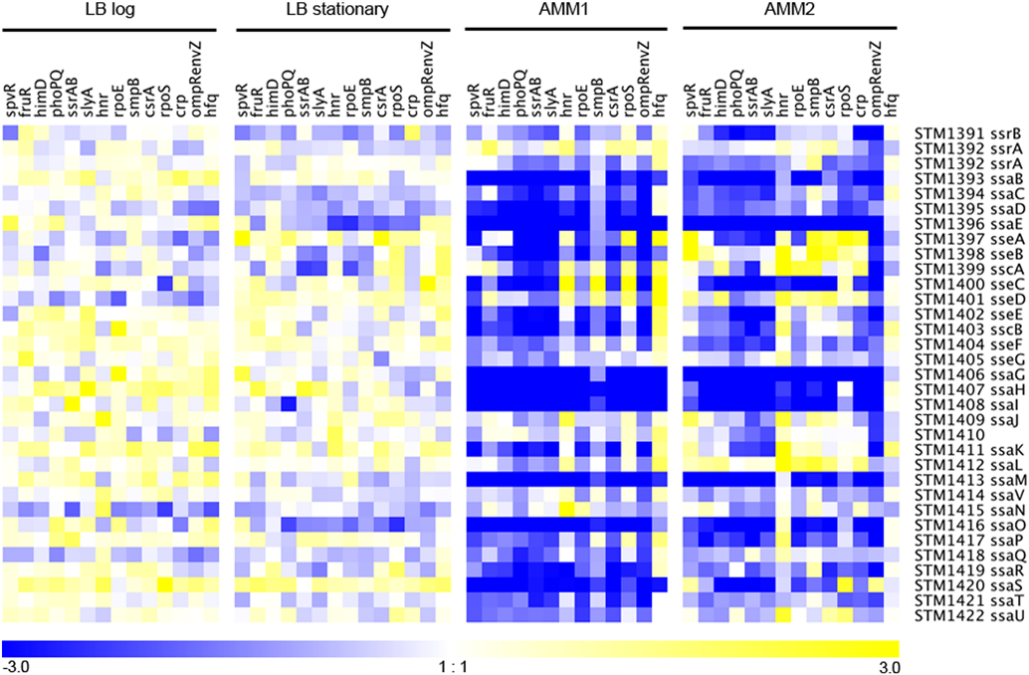
Transcriptional profiles of SPI-2 genes encoding components of the type III secretion apparatus. In each case we have computed the ratio of the microarray results for a given gene in a specific mutant background to the results of the parental strain grown under identical conditions. The results were determined under four different growth conditions (from left to right rich medium (LB) logarithmic phase, LB stationary phase, acidic minimal medium 1 (AMM1) and AMM2). Rows correspond to individual genes located within SPI-2 while columns represent regulator mutants. A *crp* deletion was excluded from transcriptional profiling for AMM1 because it does not grow under this condition. Blue represents a decrease in the mutant and yellow an increase using the computer program Genesis for display (log_2_ values; [94]). Thus blue indicates genes that are positively regulated (i.e. the level of expression decreases in the corresponding mutation compared to the parent). The results show that the 14 virulence regulators under study activate expression of most genes located in SPI-2 but the effect is only observed when bacteria are grown in minimal acidic media.

Because some of the Typhimurium regulator mutants survived so poorly within macrophages that preparing mRNA from intracellular bacteria was not possible we have used *in vitro* growth conditions that duplicate some of the intracellular conditions (low pH, minimal media; [47],[48]). The 14 virulence regulators, whose absence was identified by attenuated virulence *in vivo* in this study, presumably sense specific environmental signals within the host and respond by expressing the appropriate complement of virulence factors. However the specific environmental signals are not known and therefore each strain was grown in four different conditions; to log phase or stationary phase in Luria-Bertani (LB) broth and in a low pH/low magnesium, minimal medium [47],[48]. Two different minimal media conditions were used depending only on pre-growth conditions as described in the Materials and Methods (called acidic minimal media (AMM)1 and AMM2 here); these conditions are identical to conditions previously reported for expression of SPI-2; [47],[48]. The fate of intracellular Typhimurium is determined by pre-growth conditions prior to macrophage infection and for that reason we used both conditions AMM1 and 2 [49]. To identify global transcriptional changes for each regulator, mRNAs were isolated from each strain grown under the four conditions, converted to cDNA, and used to probe a spotted non-redundant pan-*Salmonella* orf microarray using hybridization of total genomic *Salmonella* DNA as an internal control [50]. Each microarray was probed with RNA from the parent or an isogenic deletion of one of the regulator mutants prepared from bacterial cells grown under one of the four conditions. In all there were 4 biological replicates of the parent strain but one for each mutant under each growth condition. Transcriptional profiles were significantly different from the parent for known virulence factors required during systemic infection, but only if cells were grown in acidic minimal media (AMM1 or AMM2; see Figure 3; complete microarray data available at http://www.ohsu.edu/microbiology/heffron/r01.html). Z-scores ((score−mean)/standard deviation) were computed for each gene in the Typhimurium genome in each mutant background under each growth condition (Table S2). Statistical analysis of the complete transcriptional data for gene expression in AMM1 identified at least 237 genes that were reduced 4-fold or more in common comparing the mutations to the parent strain (at least 3 standard deviation based on the technical replicates). Because transcription is reduced in the mutant compared to the parent, it suggests that normally the regulator activates transcription of these genes as is shown dramatically in Figure 3 for all SPI-2 encoded virulence genes. Conversely, only 45 genes showed a 4-fold or more up-regulation in the mutant backgrounds when cells were prepared under the same growth condition, suggesting that the regulator normally represses these genes. A more precise statistical analysis of genes that are co-regulated with SPI-2 is provided below (Table 2). All in all, these results suggest that the normal function of each of the 14 regulators is to activate transcription of virulence factors necessary for systemic infection but only under specific environmental conditions. Acidic minimal media (AMM1) provides the best induction condition of those that were examined.

**Table 2:** Genes co-regulated with the SPI-2 type III secretion system as determined by cluster analysis.

Category	No.	%	Genes co-regulated with SPI-2
Translation, ribosomal structure and biogenesis	4	3.3	STM1548, STM1549, *rpsS*, STM4508
RNA processing and modification	0	0	
Transcription	6	4.9	*leuO*, STM0859, *lrp*, STM1547, STM2748, *rob*
Replication, recombination and repair	0	0	
Cell cycle control, cell division, chromosome partitioning	0	0	
Defense mechanisms	2	1.6	*yadG*, *ydhE*
Signal transduction mechanisms	1	0.8	STM1697
Cell wall/membrane/envelope biogenesis	5	4.1	*tsx*, *dacC*, *ybjR*, *pdgL*, *yciB*
Cell motility	0	0	
Extracellular structures	0	0	
Intracellular trafficking and secretion	0	0	
Posttranslational modification, protein turnover, chaperones	0	0	
Energy production and conversion	1	0.8	*napB*
Carbohydrate transport and metabolism	4	3.3	STM0860, *pagO*, STM3251, *rbsD*
Amino acid transport and metabolism	10	8.1	*leuC*, *leuB*, *brnQ*, *ycaM*, *potC*, STM1636, *hisH*, *hisA*, *cysM*, *pepA*
Nucleotide transport and metabolism	1	0.8	*allA*
Coenzyme transport and metabolism	0	0	
Lipid transport and metabolism	0	0	
Inorganic ion transport and metabolism	1	0.8	*sodC*
Secondary metabolites biosynthesis, transport, and catabolism	1	0.8	*osmC*
General function prediction only	3	2.4	STM1097, STM1676, *yqfA*
Function unknown	7	5.7	*yafK*, STM0884, STM1632, STM2336, STM2780, STM3516, STM4549
Not in COGs	40	32.5	*pefI*, *bcfD*, STM0081, STM0082, STM0306, STM0307, *yaiA*, STM0972, STM1029, *pagD*, STM1276, *osmE*, STM1485, STM1540, *pqaA*, STM1553, STM1561, STM1600, *ugtL*, *yncJ*, STM1630, *ycjE*, *osmB*, *yciE*, STM1864, STM1941, STM2138, STM2139, STM2242, STM2245, STM2287, *pagK*, STM2778, STM2779, STM3156, *yhcO*, STM3527, *fidL*, *lpxO*, *mopB*
SPI-2	36	29.3	
Signal trtansduction			*ssrA*, *ssrB*
Intraceellular trafficking and secretion			*ssaC*, *ssaV*, *ssaN*, *ssaQ*, *yscR*, *ssaX*, *ssaT*, *ssaU*
Cell motility			*ssaJ*
General function prediction only			*sscA*, *sscB*
Not in COGs			*sifA*, *ssaB*, *ssaD*, *ssaE*, *sseA*, *sseB*, *sseC*, *sseD*, *sseE*, *sseF*, *sseG*, *ssaG*, *ssaH*, *ssaI*, STM1410, *ssaK*, *ssaL*, *ssaM*, *ssaO*, *ssaP*, *sifB*, *sseJ*, *steC*
SPI-3	1	0.8	*pipB*
Total	123	100	

The CLR edge strengths (as z-scores between pairs of genes using a cutoff of 5 standard deviations) were used as a distance matrix for hierarchical clustering [20]. Gene pairs which had no edge above the threshold indicated above were assigned a 0. The clusters were chosen at a maximum separating distance (between clusters) of 0.02 and 0.015 for the regulator and GSE2456 networks respectively. These values were chosen using the elbow criterion choosing the minimum number of clusters that explains the maximum amount of variance in the data. This was performed for each network and a cluster that contained the SPI-2 genes was identified.

Genes were grouped into categories based on known function listed on Clusters of Orthologous Groups (COGs) in NCBI.

### Validation of SPI-2 regulation

**Figure 4:**
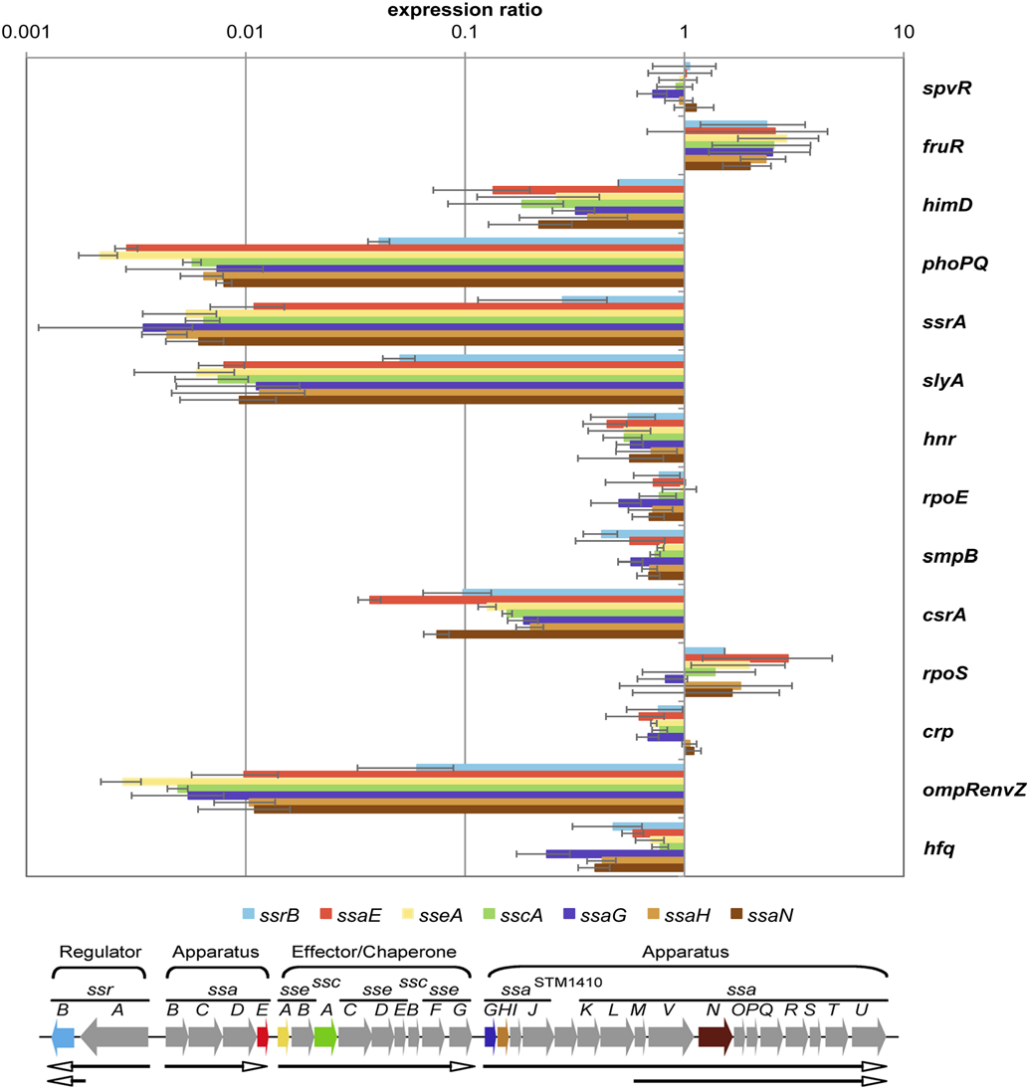
Validation of microarray results by qRT-PCR for 7 genes located within SPI-2. The bottom part of the figure shows a map of the type III secretion system located within SPI-2 with the genes that were quantified colored the same as the corresponding bars above. The top part of the figure shows expression of 7 SPI-2 genes in each regulator mutant in comparison to the parental strain when grown in AMM1. The results are plotted on a logarithmic scale. Values were normalized using *gyrB* mRNA level and represent the average of RNA prepared from three independent biological samples. The results show that mutations in 6 regulators (*himD*, *phoP*/*phoQ*, *ssrA*/*ssrB*, *slyA*, *csrA*, and *ompR*/*envZ*) strongly decrease expression of SPI-2, in 5 strains there is a significant decrease (*hnr*, *rpoE*, *smpB*, *crp*, *hfq*) while in the remaining 3 no decrease or even an increase is observed for *fruR* (the others showing no change are *spvR* and *rpoS*).

SPI-2 encodes a type III secretion system and secreted effectors required for systemic mouse infection [8],[9]. To assess the effects of growth conditions on expression of the SPI-2 secretion apparatus we constructed a *lacZ* transcriptional fusion to *ssaG*, a component of the secretion apparatus, and tested expression in each mutant background under each of the four growth conditions. At the same time we determined transcript levels via quantitative real-time PCR (qRT-PCR), using transcripts from *rpoD* and *gyrB* as controls [51]. We observed that the results determined by these two methods matched closely (Figure S2) and that the level of transcription of *ssaG* was highest when *Salmonella* was grown in minimal acidic media. In agreement with the microarrays, the effect of these regulators on *ssaG* expression was minimal if the bacteria were grown in rich media (LB broth). Because the type III secretion system and associated virulence factors encoded within SPI-2 were most highly expressed in acidic minimal media we therefore focused on growth in this media and used qRT-PCR to measure transcript levels. To validate the transcriptional profiles we prepared RNA from mutants and parent bacteria grown in acidic minimal medium and used as template for qRT-PCR. Six promoters have been identified for the type III secretion system encoded within SPI-2 (see Figure 4; [52]). We monitored transcription of seven genes within SPI-2, covering each operon at least once, and used *gyrB* transcript as an internal control. We observed a decrease in transcription of all 7 genes in 11 of the 14 mutants. The three exceptions were *spvR*, *fruR*, and *rpoS*. Strains containing mutations in *phoP*/*phoQ*, *ssrA*/*ssrB*, *slyA*, and *ompR*/*envZ* showed at least 100-fold decreases in transcription of all SPI-2 genes (Figure 4). Mutations of *ihf* (*himD*) and *csrA* showed an intermediate level of 8-16-fold decreased transcription whereas *hnr, rpoE, smpB, crp and hfq* showed a modest decrease of 2-8-fold. The results of this analysis are generally concordant with the microarray results although the dynamic range was larger for qRT-PCR than for the microarrays as has been observed before [53]. Note that *rpoE* and *hfq* mutants showed a dramatic reduction in macrophage survival, but little difference in transcription of SPI-2 genes during growth in AMM. It has recently been reported that the translational regulator Hfq, regulates translation of RpoE explaining in part why the two mutations behave similarly [54]. Furthermore, Hfq regulates translation of more than 20% of all *Salmonella* proteins explaining why a mutation in this gene has such a dramatic phenotype ([54]; Charles Ansong, Hyunjin Yoon, Steffen Porwollik, Heather Mottaz-Brewer, Briana Ogata-Petritis, Navdeep Jaitly, Joshua N. Adkins, Michael McClelland, Fred Heffron, and Richard D. Smith; Global systems-level analysis of small RNA-mediated translational regulation: Implications for virulence and global protein translation; Submitted).

### Cluster analysis identifies additional genes that are co-regulated with known virulence factors

**Figure 5:**
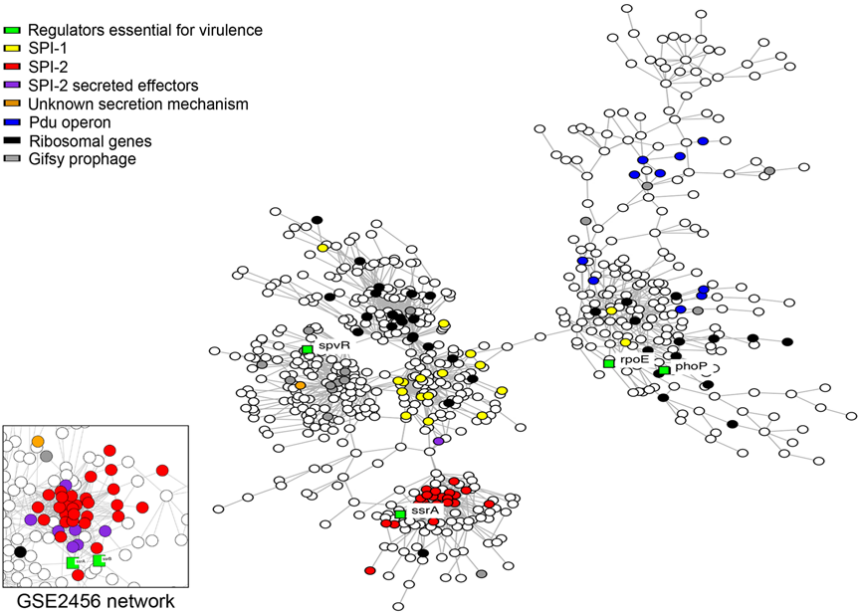
Co-clustering of genes showing similar patterns of regulation. The expression profiles for all genes were input to SEBINI [18] and analyzed using the CLR algorithm [19]. The results shown in the figure use a force-directed network layout algorithm where genes (shown as small colored circles) are generally closer together when their statistical association, and thus degree of predicted co-regulation, is stronger (the cutoff for the genes shown is 5 standard deviations from the mean or greater; p<.0001). The results were visualized as a network of similarity relationships using Cytoscape [57]. Insert cluster shows the same analysis but on a different data set derived from the NIH sponsored gene expression omnibus GEO (cutoff score 5). Co-clustered genes are described in Table 2.

The results from the microarray analysis identified many genes that appeared to be coordinately regulated including all SPI-2 encoded genes that are part of the type III secretion system (Table S2). We searched for additional previously unidentified virulence factors encoded elsewhere on the chromosome that show the same pattern of expression as those located within SPI-2. A systematic way of identifying co-regulation is with one of several computer algorithms that group genes together if their expression is similarly changed under like conditions. The diverse growth conditions used in this study, as well as differences between isogenic strains containing mutations in virulence regulators, make our data set ideal for this type of analysis. In addition, we also performed cluster analysis using public data sets acquired from the NIH sponsored public repository of microarray data, gene expression omnibus (GEO). We chose 120 transcriptional profiles from GEO that were derived by extracting RNA from the same parent *Salmonella* strain grown under a wide variety of culture conditions (GSE2456; G. Yun *et al.*, unpublished data). Expression profiles for all genes were uploaded to SEBINI (Software Environment for Biological Network Inference [18]). SEBINI incorporates statistical and network algorithms into a framework for network inference [55],[56]. To detect dependencies among genes over different conditions we employed the context likelihood of relatedness algorithm (CLR; [19]), which is a plugin for SEBINI. The results were visualized as a network of similarity relationships using Cytoscape [57]. A recent study with *E. coli* shows that the CLR algorithm was the most accurate in computing the correct network for experimentally verified regulatory interactions [19]. The results shown in Figure 5 use a force-directed network layout algorithm where genes (shown as small colored circles) are generally closer together when their statistical association, and thus degree of predicted co-regulation, is stronger (the cutoff for the genes shown is 5 standard deviations from the mean or greater; p<.0001). All SPI-2 secretion apparatus genes as well as associated effectors and chaperones were found to very tightly cluster in the network. In support of this conclusion, the cluster analyses based on our data and based on publicly available data sets are consistent. The cluster analysis identified 92 genes that are co-regulated with the SPI-2 encoded type III secretion system but not located within SPI-2. This includes known SPI-2 secreted effectors encoded elsewhere on the chromosome as well as many genes for which no function has been assigned (all total 123 genes; see Table 2). Several of these genes are A+T rich (>60% compared to 48% for the *Salmonella* chromosome) and located within sequences that are not present in close relatives of *Salmonella* suggesting that they may be unidentified secreted effectors or other virulence determinants that have been acquired from other pathogens. All in all, the implication of the cluster data is that the genes shown in Table 2 are co-regulated with SPI-2. One interesting point is that the algorithm does not distinguish between positive and negative regulation only that it is coincident. We analyzed expression of each of the 123 genes and found that only two genes (*pepA* and *mopB*) are negatively regulated; the remaining 121 are positively regulated.

### Computer aided analysis of the regulatory network

**Figure 6:**
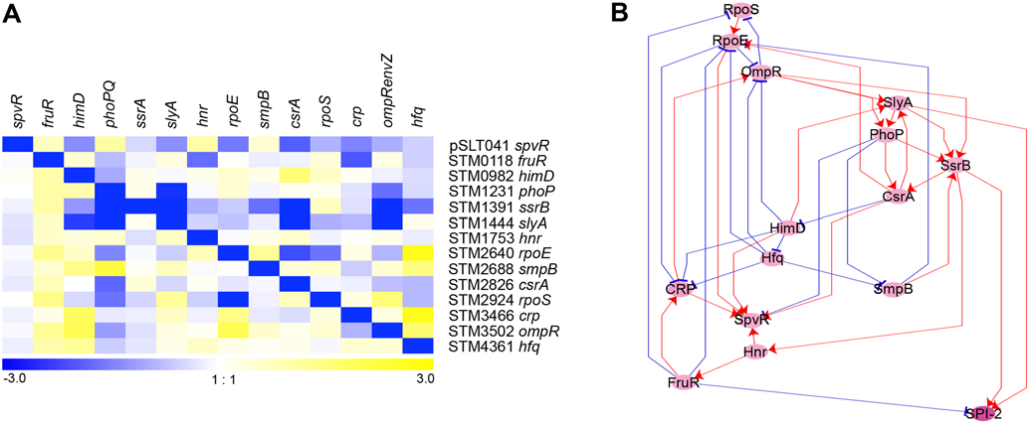
A model for regulatory interactions. A. Transcription of all 14 regulatory genes in each regulator mutant background was determined by qRT-PCR using RNA isolated from bacteria grown in AMM1. Horizontal axis represents the genes under study; vertical represents an in-frame deletion of the regulator indicated. Values shown were normalized to *gyrB* mRNA level. Color intensity represents an average expression ratio between mutant and parent in a log_2_ scale from three independent RNA samples; blue and yellow blocks indicate activation and repression by corresponding regulators respectively. Results are displayed in Genesis (log_2_) [94]. B. Cytoscape image of the hierarchical order of regulation under SPI-2 inducing conditions was constructed based on qRT-PCR data as described in Lee *et al.* [59]. Nodes indicate regulators or SPI-2 and red and blue arrows indicate activation and repression described as “edges”. Six SPI-2 genes (*ssaE*, *sseA*, *sscA*, *ssaG*, *ssaH*, and *ssaN*) were used in the matrix construction and depicted as one node in the network for the simplicity. The arrows or edges do not distinguish between direct and indirect effects except as determined experimentally. A similar regulatory hierarchy was predicted using the CLR algorithm data and matrix analysis data based on all of the expression data and is included as Figure S3.

The fact that all of the 14 regulators in this study have biological functions during infection suggests they could be part of a coordinated network. Acidic minimal medium is a well-established *in vitro* condition for SPI-2 expression; accordingly, we focused on examining regulation of genes expressed under these conditions [47],[48],[58]. To identify coordinated regulation we compared expression of each regulator in each mutant background when grown in minimal acidic medium (Figure 6A). RNA samples were prepared from three separate cultures and used as a template in separate qRT-PCR experiments. A matrix with 14 mutants in columns and 14 regulatory genes and SPI-2 genes (6 genes except for *ssrB* as used in Figure 4) in rows was constructed based on qRT-PCR data and z-scores were calculated based on average and standard deviations from columns and rows. A network among regulators and SPI-2 was mapped as described in Lee *et al.* by sorting out values that changed in a specific mutant background [59]. We visualized the resulting relationships using Cytoscape [57]. Nodes indicate regulators or SPI-2 and red and blue arrows indicate activation and repression respectively (see Figure 6B). In the computed network multiple regulators act both directly and indirectly to control SPI-2 expression. However, direct or indirect regulation cannot be distinguished without additional experimental verification. So, in Figure 6B all regulatory effects on SPI-2 have been removed except for those mediated directly by *slyA* and *ssrB* based on additional genetic data as described below. The network suggests that both *slyA* and *ssrA*/*ssrB* could coordinate regulation of SPI-2 and other virulence factors by integrating signals from multiple regulators as was tested next. An overall network was also generated by integrating 4 data sets; two CLR algorithm data from the complete microarray results and GSE2456 public microarray database and two matrix analysis data from the transcription profiles and qRT-PCR results (Figure S3; the limitation is that no distinction is made between positive and negative regulation in CLR algorithm data). The consensus network combining all data reported in this study includes the network computed from qRT-PCR (Figure 6B) in part and suggests a predictive regulatory cascade that merits a test.

### SsrB can complement other regulators of SPI-2 transcription

**Figure 7:**
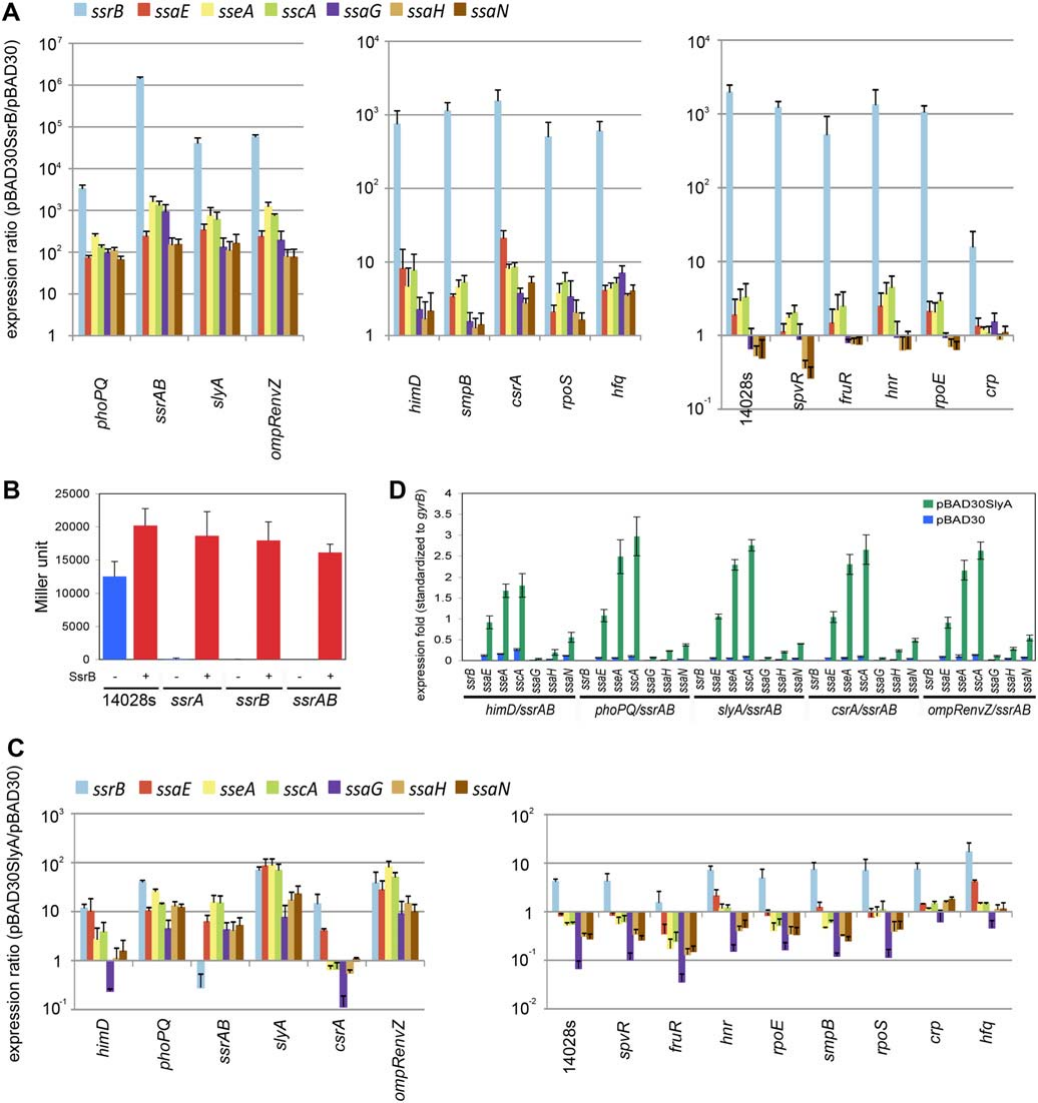
Determination of epistatic relationships among the regulators. A. To determine if expressing *ssrB in trans* can compensate for mutations in the 14 regulators we monitored transcription of 7 SPI-2 genes by qRT-PCR (triplicate biological samples, normalized results shown in each case compared to the empty vector control). Each strain containing a specific regulator deletion was transformed with pBAD30SsrB or pBAD30 and grown under AMM1 condition in the presence of 0.02% L-arabinose for 4 hours. The results are presented as expression ratio comparing the strain that over-expresses *ssrB* to the empty vector control displayed in a logarithmic scale. The values shown are averages from three separate RNA samples and grouped into three parts based on the magnitude of the effect. The results show that *ssrB* transcription is sufficient to up regulate transcription of these 7 SPI-2 genes in each mutant background. The magnitude of the effect varied from several hundred-fold to 2-fold for *crp*. B. *ssrB* was expressed in strains missing the response regulator (*ssrB*), the signal sensor (*ssrA*) or both and ß-galactosidase expression was monitored using P*_ssaG_*::*lacZ* (pFssaGTC; see Materials and Methods for details). The results show that *ssrA* is not necessary for *ssrB* to induce transcription of downstream genes (in this case *ssaG*) at least when *ssrB* is over-expressed. C. Complementation with pBAD30SlyA was performed in the same way as in Fig. 7 A in the 15 strains being investigated. The results show that transcription of *slyA* can suppress mutations in the other regulators although the results are strongest for *himD*, *phoP*/*phoQ*, *ssrA*/*ssrB*, and *ompR*/*envZ*. For all other regulators the effect of over-expression of *slyA* varied between the five SPI-2 encoded promoters and the genes that they express. D. *slyA* expression increases transcription of some SPI-2 genes even when *ssrB* is deleted. SsrB-independent SlyA activation on SPI-2 was further tested by *slyA* expression in double deletions of *himD*/*ssrAB*, *phoPQ*/*ssrAB*, *slyA*/*ssrAB*, *csrA*/*ssrAB*, and *ompRenvZ*/*ssrAB*. Expression fold compared to the parent strain harboring the empty vector is displayed. The results show a dichotomy for SlyA-mediated transcription of different operons located within SPI-2; a pronounced effect is observed for operons that encode *ssaB-E* and *sseA-F* but not for the two that encode the major structural components *ssaG-U*.

SsrB binds within SPI-2 and activates SPI-2 genes for transcription. However the location of binding sites varies suggesting that SsrB regulation is unusual [52]. Some of the 14 regulators in this study have been previously shown to directly regulate *ssrA*/*ssrB*; OmpR binding to P*_ssrA_*_/*ssrB*_ [60],[61]; SlyA binding to P*_ssrA_* [62]; PhoP binding to P*_ssrB_* [45]. To distinguish direct from indirect effects, we carried out epistasis experiments and determined if *ssrB* expression could suppress the phenotype of the other regulators of SPI-2 expression. Each isogenic derivative was transformed with either pBAD30SsrB or the empty vector control. We investigated the same seven SPI-2 genes as in the qRT-PCR assays above, and results are shown in Figure 7A [52]. Expression of an episomal copy of *ssrB* resulted in expression of SPI-2 genes in each mutant background, suggesting that SsrB is epistatic to other regulators for SPI-2 transcription. This result is in agreement with the model shown in Figure 6 where all positive regulation takes place via either *ssrB* or *slyA* or both as tested further below. Furthermore, SsrB could complement all three deletions Δ*ssrB*, Δ*ssrA*, and Δ*ssrA*/*ssrB* (Figure 7B), suggesting that over-expression of SsrB may compensate for differences in phosphorylation that normally play a role in regulation [52].

### SlyA can complement other regulators for SPI-2 expression

The number of inputs at each regulator shown in Figure 6B corresponds to the number of regulators that directly or indirectly control its expression. The number of regulators that act on *slyA* was surprising. Quantitative RT-PCR confirmed that *slyA* transcription was reduced in 11 of 14 mutant backgrounds (Figure 6A). *slyA* transcriptional activation by PhoP has been reported [63],[64]. The complementation result suggests that many of the regulators may function on SPI-2 through SlyA activation of *ssrB* or alternatively via both regulators (*slyA* and *ssrB*) acting together. To determine epistatic relationships, we introduced a *slyA*-expressing plasmid, pBAD30SlyA, into each regulatory mutant and examined complementation in each mutant background compared to an empty vector control (Figure 7C). The construct strongly complemented *ompR*/*envZ*, *phoP*/*phoQ*, *csrA* and *himD*, suggesting that the effect of these regulators on SPI-2 expression may be indirect via regulation of *slyA*. Surprisingly, the expression of SPI-2 genes depended upon *slyA* even in an *ssrA*/*ssrB* deleted strain. This suggests that *slyA* can regulate expression of SPI-2 in a way that is independent of *ssrB*. To test this possibility we constructed double deletions of *ssrAB/ompRenvZ*, *ssrAB/phoPQ*, *ssrAB/csrA* and *ssrAB/himD*. As shown in Figure 7D, *slyA* is capable of activating expression of SPI-2 genes independently of *ssrB*, *ompR/envZ*, *phoP/phoQ*, *csrA* and *himD* (*ihf*), although the effects are not as strong as *ssrB*. Furthermore there was a clear dichotomy in expression between the first two SPI-2 operons encoding *ssaB*-*ssaE* and *sseA*-*sseG* and the operons further downstream (*ssaG-ssaQ*) suggesting that *slyA* may act at different sites within SPI-2. However, these experiments are based on over-expression of *slyA* that may complicate the analysis because of binding to sites that would normally not be occupied.

## Discussion

### During systemic mouse infection, *Salmonella* processes multiple environmental cues via more than 14 regulators

We performed virulence assays on 83 regulators previously identified as required for *Salmonella enteriditis* virulence by one or more negative selection experiments in various hosts including calves, chickens, and mice [13], [21]–[23]. The mutant strains devoid of 83 regulators were tested in three mouse virulence assays to identify a subset of 14 that were most highly attenuated in a systemic mouse infection model. These 14 regulators are very diverse including alternative sigma factors (*rpoS* and *rpoE*), two-component regulators (*ompR/envZ*, *phoP/phoQ*, and *ssrA/ssrB*), post-transcriptional regulators (*csrA*, *hfq* and *smpB*), a bending protein (*ihf*) and an assortment of other DNA binding proteins (*fruR*, *spvR*, *crp*, *slyA*, and *hnr*; see Table 1 for references). *Salmonella* follows a short course of infection after intraperitoneal infection, as we have used here, passing through the lymph nodes in the peritoneal cavity to the blood stream and then colonizing and replicating within the spleen and liver. During this entire trip the bacteria are located within cells either neutrophiles or monocytes although at lower numbers in B and T cells and dendritic cells of every subclass [16],[37],[38],[65],[66]. Because the bacteria are exclusively intracellular we tested the hypothesis that replication in macrophages could be a surrogate for systemic mouse infection. Mutations that were attenuated for growth in primary macrophages were also attenuated in the mouse but the converse was not always true. Next, we used expression profiling to define the regulatory pathways. Transcriptional profiles from intracellular bacteria at different times after infection are likely to match most closely the environments encountered by *Salmonella* during infection. However, isolation of RNA from intracellular bacteria was not used because we wished to test a spectrum of regulatory mutants several of which simply did not survive within cells long enough to allow RNA preparation. RNA was therefore prepared using four laboratory growth conditions two of which partially mimic the intracellular environment (acidic minimal media).

We compared the transcriptional profiles we observed using laboratory growth conditions that mimic intracellular conditions, to those that have been performed during intracellular replication within J774 macrophage like cells. We computed the z-score for each *Salmonella* gene from our data and from the data provided by Eriksson *et al.* [67] thus providing a value that can be compared across different experiments. To compare the two sets of data we subtracted the z-scores computed from our data for AMM1 from that computed from expression profiles of intracellular *Salmonella*. As the expression pattern changes with time after infection we computed the difference for each time as well as an average for all three-time points reported (4, 8, and 12 hours after infection). There were 102 genes where the difference in z-score was 2 or greater, and 54 genes of 102 were annotated as putative, hypothetical, or conserved hypothetical. Four of the 5 most strongly differentially induced genes include magnesium transporters (*mgtB* and *mgtC*), an acid shock protein (STM1485), and a high affinity phosphate transporter (*pstS*) suggesting that the acidic minimal media we used may not be low enough in magnesium, phosphate, or may not be sufficiently acidic. Many genes are transcribed inside cells but may be only weakly transcribed or not at all transcribed in acidic minimal media. Examples of such genes include *sifA* and the operon STM3117–3120. This operon encodes some of the most abundantly expressed proteins by intracellular *Salmonella*. This result is striking, given that STM3117–3120 are not transcribed under a variety of *in vitro* conditions including AMM1 and 2 and the many conditions corresponding to the transcriptional profiles reported for microarrays archived in GEO ([68], J. McDermott and L Shi, Unpubl. Obs.). The nature of the inducing signal(s) that results in expression of these genes during intracellular growth is not known.

The regulatory network controlling expression of the genes necessary for systemic infection is complex. In our transcriptional network each pink node represents a regulator (Figure 6 B) and lines represent positive or negative transcriptional interactions (positive in red and negative in blue). In *E. coli* most regulation has been shown to follow one of three motifs: feedforward in which a regulator controls a second regulator, single input in which a regulator uniquely controls a set of downstream genes, and so called dense overlapping regulons in which there are multiple regulatory inputs to a single operon [69]. A feedforward loop can act as an electronic “AND-gate” preventing expression except when two or more signals are sensed as we see for *slyA* (upstream) and *ssrB* (downstream); *fruR* (upstream) and *crp* (downstream). Many of these predicted relationships have been demonstrated already but some are new and merit further investigation. The single input motif is found in systems of genes that form a protein complex such as both of the type III secretion systems in *Salmonella*. For SPI-2 *ssrB* plays this role and for SPI-1 *hilA* is the central regulator. The multiple promoters located within SPI-2 presumably respond to differences in *ssrB/slyA* activation, perhaps establishing part of the temporal order of expression following phagocytosis of *Salmonella*. There are other regulators required for systemic infection in BALB/c mice, including those whose absence reduces viability without a compensating mutation (*hns*; [70]), those that require deletion of two unlinked genes for inactivation (ppGpp; reviewed in [71] and *ydgT*/*hha* [48]), or those that were simply missed in the screens (STM0410; [72]); these regulators will be included in subsequent analyses.

### What are the signals being sensed during systemic infection and how are they integrated to express virulence factors appropriately?

There have been several studies to identify environmental factors that regulate expression of the type III secretion system encoded within SPI-2. Carbon limitation, low concentrations of Mg^2+^ or Ca^2+^ [73], and acidic pH [47],[61] induce SPI-2 expression *in vitro*. Inside professional phagocytic cells divalent cation concentrations and the presence of defensin-like molecules signal through *phoP*/*phoQ* [74],[75], and acidic pH and osmolarity signal through *ompR*/*envZ* [76]. OmpR has been shown to respond to acidic pH via *cadC* [77]. The signal(s) received by SsrA, the sensor of the SsrA/SsrB system, is not yet established. It is surprising that over-expression of *ssrB* can compensate for a deletion of *ssrA* or *ssrA*/*ssrB*. Previous results have shown that a conservative replacement of the amino acid that is the essential phosphate acceptor eliminated expression of SPI-2 genes suggesting that SsrB requires phosphorylation for activity. Yet, expression of *ssrB* without *ssrA* results in expression of all 7 SPI-2 genes examined (Figure 7A). It is possible that SsrB can be phosphorylated from other sources as noted by Walthers *et al.* [52] or that the over-expression results in dimerization and self activation similar to what has been observed for PhoP [78].

Mutations in *rpoE*, *smpB*, *rpoS*, and *hfq* showed only small decreases in SPI-2 gene transcription during growth in minimal acidic media but some of them showed large defects in survival assays performed in murine macrophages. For *rpoE* and *rpoS* it is possible that the environmental conditions that distinguish growth in minimal media from those in the SCV inside hosts are sensed via these two alternative sigma factors. Figure 6B shows that there is a close relationship between these two regulators and that they can both affect each other through the *ompR/envZ* two-component regulator. Post-transcriptional SPI-2 regulation is a likely explanation for the large intracellular growth defects observed in the mutant *smpB* and *hfq* derivatives despite relative small transcriptional effects. Strains containing mutations in *spvR* and *hnr* showed comparable survival levels to wild-type in macrophages from BALB/c mice despite the fact that they are attenuated in the same mouse strain. Perhaps these regulators are selected during growth in other cell types or within phagocytic cells that are activated as a consequence of the inflammatory response generated by the bacteria.

### SlyA and SsrA/SsrB together activate transcription of the SPI-2 encoded secretion apparatus

SlyA is a DNA-binding protein with high affinity for inverted repeat sequences [79] and the binding ability of SlyA to the *ssrA* promoter region was previously reported [62]. SlyA binds to a specific palindromic sequence, but also to other binding sites that do not fit a consensus sequence [79],[80]. Similarly SsrB binds upstream of *ssrA* in a region of the promoter typical of response regulators but has no obvious recognition site and binds within the operons that encode structural components of the type III secretion system [52],[81]. Four nucleoid-like proteins in enteric bacteria, H-NS, StpA, Hha, and YdgT have a predilection for binding to A+T rich sequences and repress transcription of SPI-2 genes (rev. in [14]). These proteins have a degenerate recognition sequence and the ability to polymerize along and bridge adjacent stretches of DNA repressing transcription apparently by occlusion of RNA polymerase. It has been shown that virulence regulator *slyA*, and close homologues *rovA* [82] and *toxT* [83] act to relieve the repression caused by H-NS at specific promoters [48],[70],[84],[85]. SPI-2 regulation by SsrB and SlyA may be explained in a similar mechanism where both SlyA and SsrB counteract the silencing activity of H-NS/YdgT/Hha by competing for binding to the same target sequences or by altering the structure of the DNA to promote transcription for SPI-2. Walthers *et al.* [52] and Feng *et al.* [81] found that there was no consensus SsrB binding site or distance relative to the transcription start sites and Walthers (ibid) proposes that different promoters may be activated by distinct SsrB mechanisms. Our results support this conclusion and extend the observation to *slyA* at least for specific SPI-2 transcripts. A proposed mechanism for activation is that SlyA competes with a repressor for binding to the promoter and subsequently facilitates RNA polymerase access [80]. This model is supported by SlyA binding sites that are located downstream of transcriptional start sites [31],[86], which is unusual for a traditional transcriptional activator. In agreement with this model, SlyA and PhoP counteract H-NS silencing at *pagC* [85],[87]. Thus, one explanation for the transcription we observe following over-expression of *ssrB* or *slyA* may be that both SlyA and SsrB counteract binding of small nucleoid-like proteins including both H-NS and YdgD/Hha in this A+T rich SPI-2 region [70]. This possibility may be reflected in the differences in expression of the five operons within SPI-2 following *ssrB* and *slyA* over-expression.

### Clustering analysis and co-expression

The complex regulatory network suggested by these studies has been partially confirmed. Complete elucidation of the virulence pathways must include proteomic studies to identify steps that require translational regulation, binding studies to distinguish direct from indirect effects and additional regulators not included in this preliminary group of 14. In this study, cluster analysis was used to identify genes that show similar expression profiles under a variety of environmental conditions and from the isogenic *Salmonella* regulatory mutants we describe. The average G+C content of the 92 genes outside of SPI-2 that show the same regulation as SPI-2 is 47% while the average for *Salmonella* is 52% (p<e^−25^). Most of these genes are not found in closely related non-pathogenic bacteria (overall comparison eliminating SPI-2 genes p<e^−15^). Thus application of the CLR algorithm identified a very interesting group of genes that are co-regulated with SPI-2 and were horizontally transferred to *Salmonella*. Analysis of microarrays and other expression data was used to construct a predictive model for how the regulators may interact and coordinate regulation within the host. A few of the predictions have been verified and more are being tested. The construction of a complete interaction network describing both the host and pathogen is a long-term goal of this and other related research efforts.

## Materials and Methods

### Bacterial strains and growth conditions

STM ATCC14028s was used as the parent for all of the constructions described. Eighty-three non-polar deletions of regulatory genes were constructed using derivatives of pKD13 (AY048744, GeneBank) designed to include a DNA ‘bar code” allowing strains to be distinguished within a mixture [24],[25]. Using these plasmids as template we prepared eighty-three PCR products with sequences homologous to target genes at the 5′- and 3′- ends of the linear fragment. Deletions were constructed as described [24], confirmed by PCR and P22 transduced into the parent STM 14028. The final non-polar deletions were constructed by providing flip recombinase *in trans* [24]. The eighty-three deletion strains are listed in Table S1. Double deletions including *himD*/*ssrAB*, *phoPQ*/*ssrAB*, *slyA*/*ssrAB*, *csrA*/*ssrAB*, and *ompRenvZ*/*ssrAB* were generated in the same way. In the construction of the *himD* knockout strain, *himD* was cloned onto pCP20 (ibid) allowing expression of flip recombinase even in an *ihf* mutant, which does not support replication of pCP20. A more detailed description of these strains will be described elsewhere.

pFssaGTC was constructed by cloning *lacZ* and the promoter for *ssaG* (P*_ssaG_*) including 450 bp of upstream sequence into pZC320 (U26464, GeneBank) via a SalI site. pZC320 was kindly provided by B. D. Jones (University of Iowa, USA) and D. Lane (CNRS, France). The primer sets for PCR amplifications of *lacZ* and P*_ssaG_* are shown in Table S3. pKG137, a derivative of pCE37 (AY061943, GeneBank) was used as template for *lacZ* PCR. For pBAD30SsrB/pBAD33SsrB or pBAD30SlyA, *ssrB* and *slyA* were amplified by PCR using the oligos listed in Table S3 and subsequently cloned into pBAD30 or pBAD33 [88]. *ssrB* and *slyA* were induced by addition of 0.02% L-arabinose to acidic minimal medium 1 (see below). To complement a *spvR* deletion, a 1.7 kb DNA fragment containing *spvR* and its promoter was PCR amplified and cloned into pWSK29 (AF016889, GeneBank) via BamHI and EcoRI. All primers used in plasmids construction are listed in Table S3. Kanamycin, chloramphenicol and carbenicillin were added at 50 µg/ml, 10 µg/ml and 100 µg/ml respectively as necessary for selection.

Bacteria were grown under four different conditions to cover expression of a large number of *Salmonella* genes. They were: log phase in Luria-Bertani (LB) medium, stationary phase in LB medium, acidic minimal media 1 (AMM1; grown in AMM media overnight, diluted 1∶100 and grown for an additional 4 hrs) and AMM2 (grown in LB to stationary phase and resuspended in the same volume of acidic minimal media [47],[48]). The latter two media partially mimic the intracellular environment of the *Salmonella* containing vacuole and differ from each other only in the pre-growth conditions [47],[48]. Because of the density of the culture and the fact that the bacteria are in stationary phase the bacteria do not grow in AMM2. The formula for AMM (pH 5.0) is 100 mM Tris-Cl, 5 mM KCl, 7.5 mM (NH_4_)_2_SO_4_, 0.5 mM K_2_SO_4_, 1 mM KH_2_PO_4_, 0.2% glycerol, 0.1% casamino acids, and 8 µM MgCl_2_.

### Mouse studies

For experimental continuity with earlier work from the laboratory, 4-6-week-old female BALB/c mice were used. All experiments carried out with mice are in accordance with the protocol of Oregon Health & Science University Animal Care and Use Committee. For our initial screening, 200 colony forming units (CFU) of *Salmonella* was administered to each mouse by intraperitoneal inoculation and mouse survival was monitored for 21 days. The isogenic parental isolate, ATCC14028, rapidly kills at this dosage but any mutant that resulted in 1 or 2 mice surviving or an increased time to death of the mouse was retested. A second test at the same dosage with 5 mice was used to further reduce the number of mutations considered and finally an i.p. LD_50_ was computed by administering 10^2^, 10^4^, and 10^6^ cfu of bacteria to groups of 3 mice. LD_50_ values were determined according to the method of Reed and Muench [89] at 1-month post infection.

### Macrophage studies

Typhimurium intracellular survival/replication assay was performed as described [90]. Bone marrow-derived macrophages (BMDM) were prepared from 5-week-old female BALB/c mice as previously described [91]. *Salmonella* was grown overnight in LB and opsonized with 10% BALB/c mice serum (Innovative Research) for 20 min [90] prior to infection. BMDM cells in 24-well plates were infected with bacteria cells at MOI 100. Infections were initiated by centrifuging the bacteria onto the cell monolayers at 1,000×g for 5 min and then plates were incubated at 37°C with 5% CO_2_ for 30 min. To remove extracellular bacteria after infection, cells were washed with PBS and incubated in Dulbecco's modified Eagle's medium (DMEM) containing gentamicin (100 µg/ml) for 1 h. After treatment with 100 µg/ml gentamicin, cells were washed with PBS and overlaid with DMEM containing 20 µg/ml gentamicin for the remainder of the experiment. To enumerate intracellular *Salmonella* cells at 30 min, 2 h, and 18 h post-infection, BMDM cells were lysed in 1% TritonX-100 in phosphate buffered saline and serially diluted lysates were plated on LB agar.

### Microarray analysis

Cells were cultivated in four conditions as described above in triplicate and lysed following the instructions of Qiagen RNeasy midi kit (Qiagen) in combination with DNase I (Qiagen) treatment. Isolated total RNA was analysed by RNA 6000 nano assay (Agilent 2100 bioanalyzer, Agilent Technologies). Because of the number of microarrays necessary for this experiment, three biological replicates of RNAs from each strain were pooled with the same amount of RNA from each sample prior to labeling. Furthermore, during growth in minimal media there were substantial differences in growth rate for some regulator mutants (*crp*, *hfq*, and *csrA*). For this reason a *crp* deletion strain was excluded from transcriptional profiling for AMM1 although expression of relevant virulence factors was analyzed for a *crp* mutant by qRT-PCR at several time points as described. A total of 55 RNA pools from 14 strains in 4 conditions were prepared. An additional 12 RNA samples were prepared from the parental strain. All were subjected to microarray anaysis according to the procesures described [50]. Total RNAs were reverse transcribed with random hexamers and Superscript II (Invitrogen) and subsequent reverse transcripts were labeled by Cy3-linked dCTP (Amersham Biosciences). Genomic DNA from 14028s was isolated using GeneElute bacterial genomic DNA kit (Sigma) and labeled with Cy5-linked dCTP (Amersham Biosciences) as reference for hybridization. A hybridiztion mixture containing the same amounts of Cy3 and Cy5-labeled probes was applied to a non-redundant pan-*Salmonella* orf array and incubated in a hybridization chamber (Corning) at 42°C overnight [50]. Each array slide has identical triplicate arrays as technical replicates. Slides hybridized with Cy3/Cy5-labled probes were scanned using ScanArray Express (Packard Bioscience, BioChip Technologies) and the fluorescent signal intensities were quantified using QuantArray software (Packard Bioscience, BioChip Technologies) and exported to Excel files. The raw data was processed in WebArray [92] for statistical normalization.

### Quantitative RT-PCR analysis

Total RNA was isolated using RNAprotect Bacteria Reagent (Qiagen), RNeasy mini kit (Qiagen), and DNase to remove residual chromosomal DNA (Qiagen) according to the manufacturer's recommendations. RNA concentration was measured using a NanoDrop ND-1000 spectrophotometer (NanoDrop Technologies, Inc.). cDNA was synthesized using the iScript cDNA synthesis kit (Bio Rad) and cDNA correspondig to 10 ng of input RNA was used as template in each real-time PCR using SYBR green reagent to detect duplex DNA product (ABI 7700, Applied Biosystems). Primers used in RT-PCR are listed in Table S3. RT-PCR reaction was carried out at 95°C for 10 min, 95°C for 15 s and 60°C for 1 min for 50 cycles. The expression ratio of each gene is the average from three independent RNA samples and was normalized to the level of *gyrB* [51],[70].

### β-Galactosidase assay

β-Galactosidase activity was assayed according to the method described previously [93]. *Salmonella* cells were grown as described above. The activity was measured in triplicate using independently grown cultures and average enzyme activities were normalized to the number of input bacteria in the assay to compute Miller units.

### Accession numbers of 14 genes investigated in this study

*spvR*, 1256197; *fruR*, 1251636; *himD*, 1252500; *phoP*/*phoQ*, 1252749/1252748; *ssrA*/*ssrB*, 1252910/1252909; *slyA*, 1252962; *hnr*, 1253272; *rpoE*, 1254163; *smpB*, 1254211; *csrA*, 1254349; *rpoS*, 1254447; *crp*, 1254989; *ompR*/*envZ*, 1255025/11255024; *hfq*, 1255887. Accession number assigned here refers to Entrez Gene ID in NCBI.

## Supporting Information

Figure S1 Complementation of an *spvR* deletion in strain 14028s restores virulence. A PCR fragment containing *spvR* and its 600 bp upstream region was cloned on pWSK29 (AF016889, GeneBank) via BamHI and EcoRI. Three *Salmonella* strains of 14028s, *spvR*, and *spvR* harboring pWSK29spvR were i.p. administered to 4 BALB/c mice respectively with dose of 200 cfu per mouse. Mice survival after *Salmonella* infection was monitored for 21 days.
(0.14 MB TIF)

Figure S2 Comparison of *ssaG* transcript level as determined by different methods. The transcript level of *ssaG* was determined in each isogenic mutant under all four growth conditions used in this study by qRT-PCR in A (*gyrB* transcript as a control). An alternative method of comparing transcription levels is shown in B in which a *lacZ* reporter was used. The construct was made on a low copy F derivative plasmid as described in methods. Values reported are for β-galactosidase assays of each strain in each growth condition and show similar patterns to those from qRT-PCR. As a measure of transcript levels we chose to use qRT-PCR because we observed differences in β-galactosidase translation that did not reflect transcription.
(0.23 MB TIF)

Figure S3 Regulatory consensus network integrating 4 data sets. To produce an overall transcriptional regulatory network that links the 14 essential regulators, we combined the two CLR algorithm data (Figure 5) from our gene profiles and GSE2456 public transcriptional profiles and two matrix analyses from our microarray data and RT-PCR data (Figure 6). We limited the interconnections (or edges) to just those involving the 14 regulators and six SPI-2 genes (*ssaE*, *sseA*, *sscA*, *ssaG*, *ssaH*, and *ssaN*) and then removed all edges with z score less than 1.5. Red and blue edges represent activation and repression respectively and green edge shows regulation detected only by CLR algorithms. The CLR algorithm predicts associated regulation based on equivalent negative or positive expression profiles so does not distinguish positive and negative regulation. Activation or repression was determined in edge predictions that included at least one matrix prediction. Line thickness indicates the number of methods contributing to the consensus; the more conserved the regulation among data sets, the thicker edge. The two-component regulatory systems including *ompRenvZ*, *phoPQ*, and *ssrAB* are referred to as single nodes because the signal sensor and response regulator occupy similar positions in the network. A similar strategy was used for the SPI-2 genes although the *slyA* results described in Figure 7 demonstrate that not all of the SPI-2 encoded operons are regulated similarly. Interconnections between the regulators and SPI-2 were reduced based on the experimental data described in the manuscript. The network was visualized in Cytoscape using a hierarchical layout algorithm.
(0.23 MB TIF)

Table S1 List of 83 regulators inferred to have a virulence function and their deletion phenotypes examined in this study for mouse virulence.
(0.13 MB DOC)

Table S2 List of genes highly co-regulated by virulence regulators in this study when grown in minimal acidic media. Genes were grouped into categories based on known function listed on Clusters of Orthologous Groups (COGs) in NCBI.
(0.06 MB DOC)

Table S3 Primers used in plasmids construction (A) and qRT-PCR (B).
(0.06 MB DOC)
