# Supplementary figures and images for: Coordinated Regulation of Virulence during Systemic Infection of Salmonella enterica Serovar Typhimurium
Source: PLoS Pathog. 2009 Feb 20;5(2):e1000306. doi: 10.1371/journal.ppat.1000306 (PMC2639726; doi:10.1371/journal.ppat.1000306)

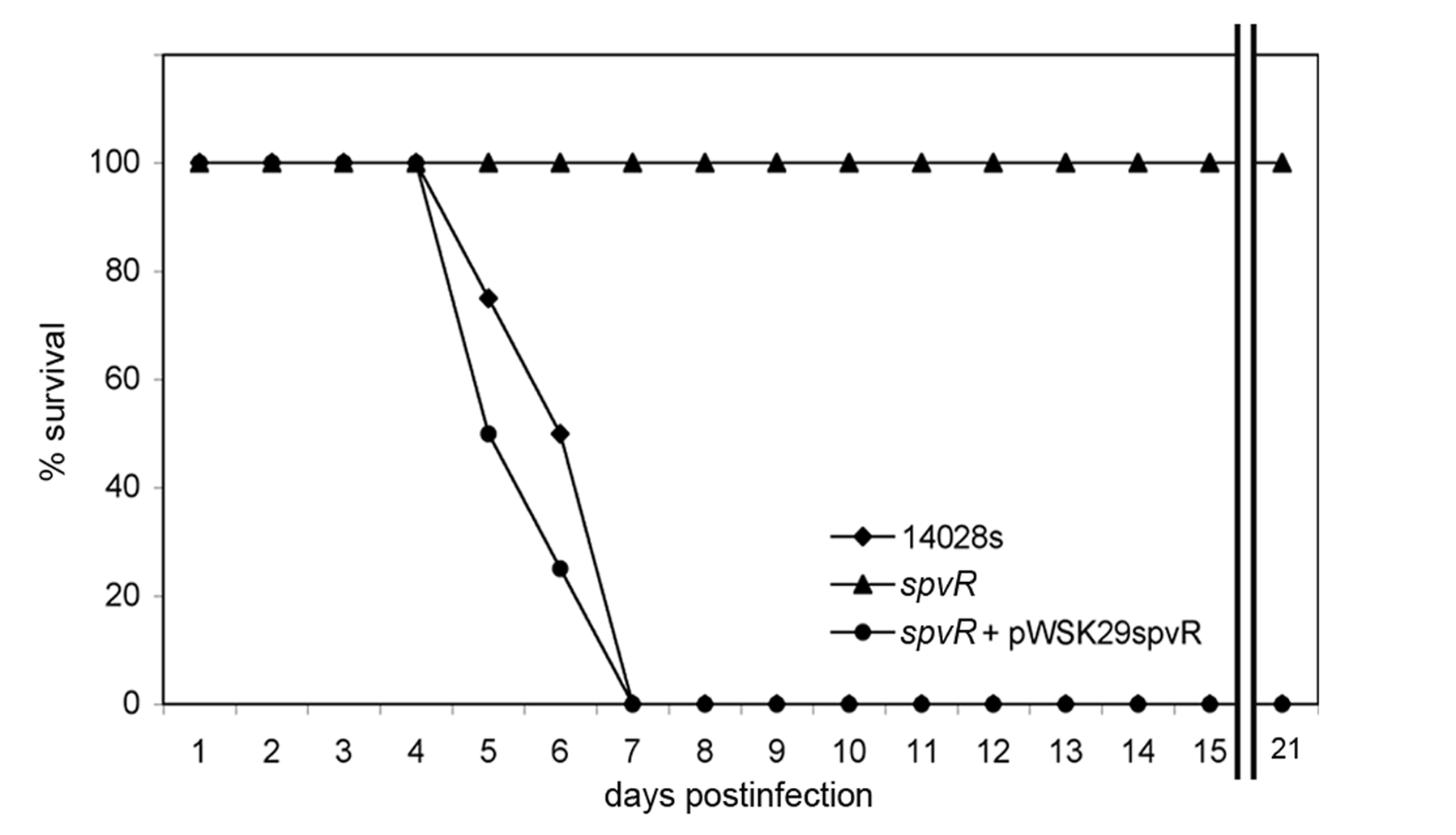

Supplement: Figure S1 — Complementation of an spvR deletion in strain 14028s restores virulence. A PCR fragment containing spvR and its 600 bp upstream region was cloned on pWSK29 (AF016889, GeneBank) via BamHI and EcoRI. Three Salmonella strains of 14028s, spvR, and spvR harboring pWSK29spvR were i.p. administered to 4 BALB/c mice respectively with dose of 200 cfu per mouse. Mice survival after Salmonella infection was monitored for 21 days. (0.14 MB TIF) [file ppat.1000306.s001.tif]

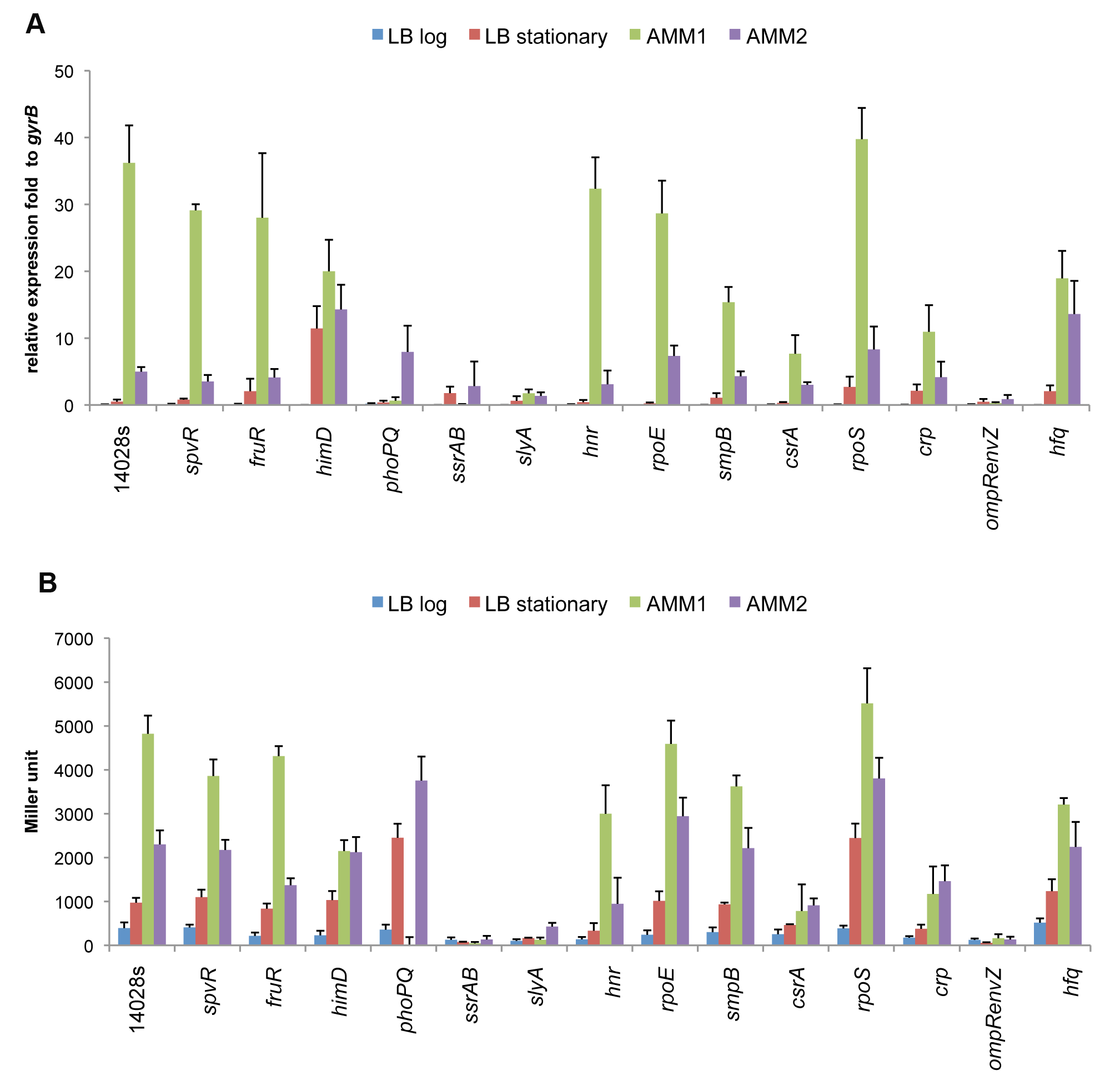

Supplement: Figure S2 — Comparison of ssaG transcript level as determined by different methods. The transcript level of ssaG was determined in each isogenic mutant under all four growth conditions used in this study by qRT-PCR in A (gyrB transcript as a control). An alternative method of comparing transcription levels is shown in B in which a lacZ reporter was used. The construct was made on a low copy F derivative plasmid as described in methods. Values reported are for β-galactosidase assays of each strain in each growth condition and show similar patterns to those from qRT-PCR. As a measure of transcript levels we chose to use qRT-PCR because we observed differences in β-galactosidase translation that did not reflect transcription. (0.23 MB TIF) [file ppat.1000306.s002.tif]

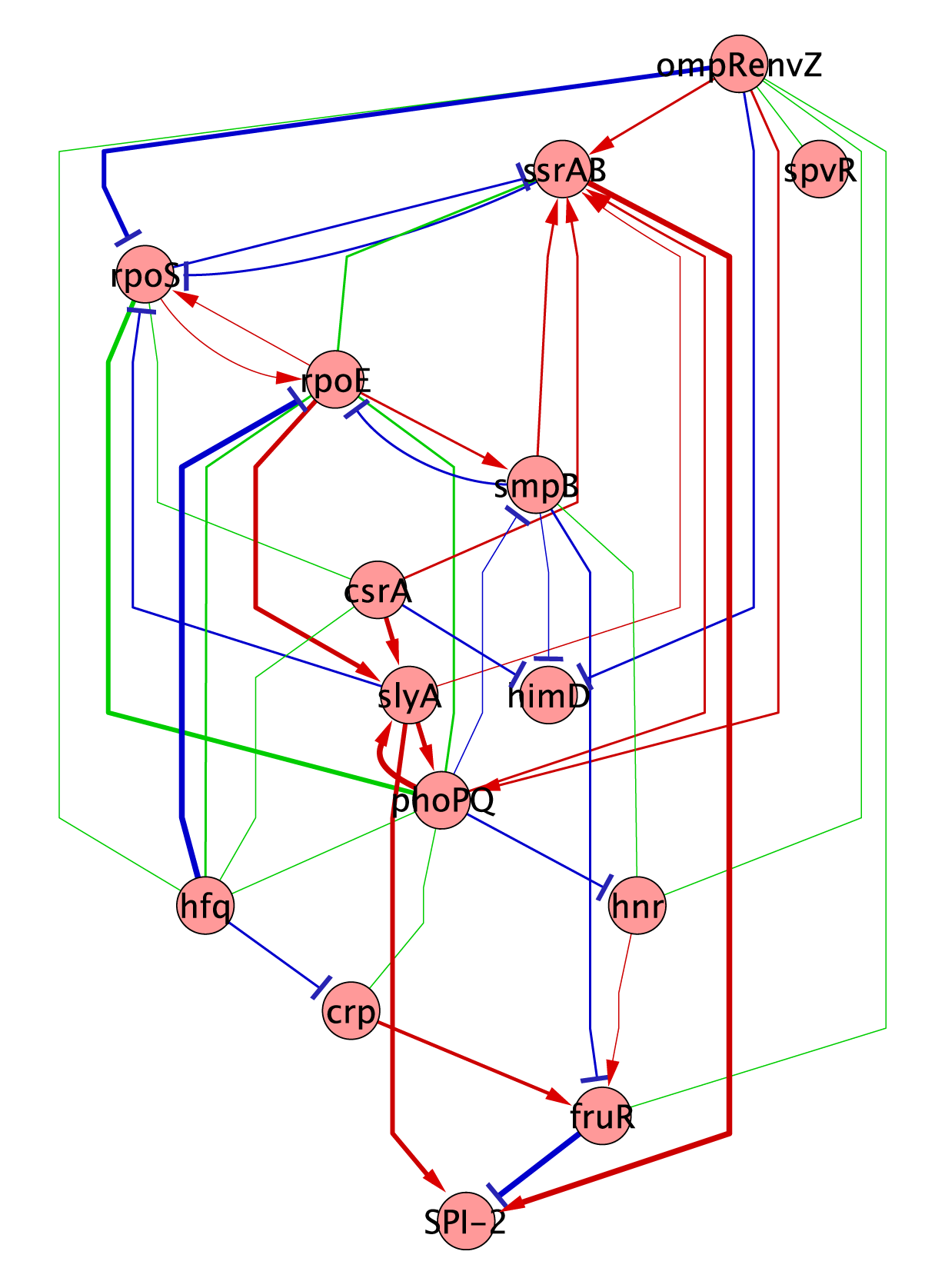

Supplement: Figure S3 — Regulatory consensus network integrating 4 data sets. To produce an overall transcriptional regulatory network that links the 14 essential regulators, we combined the two CLR algorithm data (Figure 5) from our gene profiles and GSE2456 public transcriptional profiles and two matrix analyses from our microarray data and RT-PCR data (Figure 6). We limited the interconnections (or edges) to just those involving the 14 regulators and six SPI-2 genes (ssaE, sseA, sscA, ssaG, ssaH, and ssaN) and then removed all edges with z score less than 1.5. Red and blue edges represent activation and repression respectively and green edge shows regulation detected only by CLR algorithms. The CLR algorithm predicts associated regulation based on equivalent negative or positive expression profiles so does not distinguish positive and negative regulation. Activation or repression was determined in edge predictions that included at least one matrix prediction. Line thickness indicates the number of methods contributing to the consensus; the more conserved the regulation among data sets, the thicker edge. The two-component regulatory systems including ompRenvZ, phoPQ, and ssrAB are referred to as single nodes because the signal sensor and response regulator occupy similar positions in the network. A similar strategy was used for the SPI-2 genes although the slyA results described in Figure 7 demonstrate that not all of the SPI-2 encoded operons are regulated similarly. Interconnections between the regulators and SPI-2 were reduced based on the experimental data described in the manuscript. The network was visualized in Cytoscape using a hierarchical layout algorithm. (0.23 MB TIF) [file ppat.1000306.s003.tif]
