# Supplementary material for: Coordinated Regulation of Virulence during Systemic Infection of Salmonella enterica Serovar Typhimurium
Source: PLoS Pathog. 2009 Feb 20;5(2):e1000306. doi: 10.1371/journal.ppat.1000306 (PMC2639726; doi:10.1371/journal.ppat.1000306)
Supplement: Table S1 — List of 83 regulators inferred to have a virulence function and their deletion phenotypes examined in this study for mouse virulence. (0.13 MB DOC) [file ppat.1000306.s004.doc]

**Table S1.** List of 83 regulators inferred to have a virulence function and their deletion phenotypes examined in this study for mouse virulence.

| **Gene no.** | **Gene symbol** | **i.p.** | **i.g.** | **CI** | **Gene no.** | **Gene symbol** | **i.p.** | **i.g.** | **CI** |
| --- | --- | --- | --- | --- | --- | --- | --- | --- | --- |
| **pSLT041** | ***spvR*** |  |  |  | STM2361 |  |  |  |  |
| STM0014 |  |  |  |  | STM2413 | *yfeC* |  |  |  |
| STM0030 |  |  |  |  | STM2557 | *cadC* |  |  |  |
| STM0034 |  |  |  |  | STM2575 |  |  |  |  |
| STM0052/0053 |  |  |  |  | **STM2640** | ***rpoE*** |  |  |  |
| STM0096 | *hepA* |  |  |  | **STM2688** | ***smpB*** |  |  |  |
| STM0115 | *leuO* |  |  |  | **STM2826** | ***csrA*** |  |  |  |
| **STM0118** | ***fruR*** |  |  |  | STM2866 | *sprB* |  |  |  |
| STM0237 | *rof* |  |  |  | STM2867 | *hilC* |  |  |  |
| STM0430 | *phnR* |  |  |  | STM2875 | *hilD* |  |  |  |
| STM0550 | *fimY* |  |  |  | STM2876 | *hilA* |  |  |  |
| STM0604 | *ybdM* |  |  |  | STM2912 |  |  |  |  |
| STM0606 | *ybdO* |  |  |  | **STM2924** | ***rpoS*** |  |  |  |
| STM0634 | *ybeF* |  |  |  | STM2958 | *barA* |  |  |  |
| STM0764 |  |  |  |  | STM3031 |  |  |  |  |
| STM0859 |  |  |  |  | STM3096 | *yqgE* |  |  |  |
| STM0959 | *lrp* |  |  |  | STM3124 |  |  |  |  |
| **STM0982** | ***himD*** |  |  |  | STM3245 | *tdcA* |  |  |  |
| STM1001 |  |  |  |  | STM3385 | *fis* |  |  |  |
| STM1003 |  |  |  |  | **STM3466** | ***crp*** |  |  |  |
| STM1095/1096 | *copS/copR* |  |  |  | **STM3501/3502** | ***envZ/ompR*** |  |  |  |
| STM1142 | *csgD* |  |  |  | STM3547 |  |  |  |  |
| **STM1230/1231** | ***phoQ/phoP*** |  |  |  | STM3670 |  |  |  |  |
| STM1266 |  |  |  |  | STM3736 |  |  |  |  |
| STM1315 | *celD* |  |  |  | STM3759 | *marT* |  |  |  |
| STM1355 | *ydiP* |  |  |  | STM3778 |  |  |  |  |
| STM1382 | orf408 |  |  |  | STM3785 |  |  |  |  |
| **STM1391/2** | ***ssrB/ssrA*** |  |  |  | STM4005/4006 | *glnG/glnL* |  |  |  |
| STM1430 | *purR* |  |  |  | STM4058/4059 | *cpxA/cpxR* |  |  |  |
| **STM1444** | ***slyA*** |  |  |  | STM4127 | *yijC* |  |  |  |
| STM1519 | *marA* |  |  |  | STM4165 | *rsd* |  |  |  |
| STM1660 | *fnr* |  |  |  | STM4291/4292 | *basS/basR* |  |  |  |
| STM1671 |  |  |  |  | STM4297 | *melR* |  |  |  |
| STM1704 | *yciT* |  |  |  | STM4315 |  |  |  |  |
| **STM1753** | ***hnr*** |  |  |  | STM4317 |  |  |  |  |
| STM1955 | *fliZ* |  |  |  | STM4322 | *yjdC* |  |  |  |
| STM2036 | *pocR* |  |  |  | **STM4361** | ***hfq*** |  |  |  |
| STM2159 | *yehU* |  |  |  | STM4547 | *yjjQ* |  |  |  |
| STM2269/2270 | *yojN/rcsB* |  |  |  | STM4548 | *bglJ* |  |  |  |
| STM2275 |  |  |  |  | STM4558 | *rimI* |  |  |  |
| STM2281 |  |  |  |  | STM4598 | *arcA* |  |  |  |
| STM2331 | *yfbQ* |  |  |  |  |  |  |  |  |

* Attenuated strains by i.p. and i.g. in BALB/c and competitive index in 129X1/SvJ mice are marked in grey. Genes analyzed in this study are indicated in bold.
