# Supplementary material for: Coordinated Regulation of Virulence during Systemic Infection of Salmonella enterica Serovar Typhimurium
Source: PLoS Pathog. 2009 Feb 20;5(2):e1000306. doi: 10.1371/journal.ppat.1000306 (PMC2639726; doi:10.1371/journal.ppat.1000306)
Supplement: Table S2 — List of genes highly co-regulated by virulence regulators in this study when grown in minimal acidic media. Genes were grouped into categories based on known function listed on Clusters of Orthologous Groups (COGs) in NCBI. (0.06 MB DOC) [file ppat.1000306.s005.doc]

**Table S2.** List of genes highly co-regulated by virulence regulators in this study when grown in minimal acidic media. Genes were grouped into categories based on known function listed on Clusters of Orthologous Groups (COGs) in NCBI.

| **Category** | **No.** | **%** | **Negative regulation** | **Positive regulation** |
| --- | --- | --- | --- | --- |
| Translation, ribosomal structure and biogenesis | 38 | 13.5 | *rpmE2, tufA* | *rpsB, rpsA, rmf, rmpF, rpmI, rplT, tyrS, speG,* STM1549*, prfA, rplS, rimM, rpmA, rplU, rpsI, rplM, rplQ, rpsM, rpmJ, rpmD, rpsE, rplR, rpsH, rpsN, rplN, rpsQ, rpmC, rplV, rpsS, rplW, rpsL, trpS, rpmH, rplA, rpsF, rpsR* |
| RNA processing and modification | 0 | 0 | *-* | *-* |
| Transcription | 13 | 4.6 | *rpoS* | *nusB, rnk, lrp, slyA, ydfH, rnb, fadR, cspC,* STM3084*, rpoA, cspA, yjeB* |
| Replication, recombination and repair | 7 | 2.5 | *-* | *seqA, himD, ycfH, himA, ntpA, recO, hupA* |
| Cell cycle control, cell division, chromosome partitioning | 1 | 0.4 | *-* | *ygfE* |
| Defense mechanisms | 0 | 0 | *-* | *-* |
| Signal transduction mechanisms | 6 | 2.1 | *-* | *dksA, ydiV, proQ, yecG, fliY, csrA* |
| Cell wall/membrane/envelope biogenesis | 17 | 6.0 | *manC* | *hlpA, rlpB, pal, lolA, nlpC, slyB, pdgL, yciB, yeeZ, udg, rfbC, rfbA, rfbD, galF, nlpB, smpA* |
| Cell motility | 0 | 0 | *-* | *-* |
| Extracellular structures | 0 | 0 | *-* | *-* |
| Intracellular trafficking and secretion | 6 | 2.1 | *-* | *tolR, yscR, ssaC, ssaS, ssaT, secB* |
| Posttranslational modification, protein turnover, chaperones | 14 | 5.0 | *ybeV, clpA, hflK* | *htrA, yeaA, sufA, sufC, gst, sohB, hypC, grxC, trxA, hslV, mopB* |
| Energy production and conversion | 14 | 5.0 | *fixC, adhE, pckA, glpD, atpB, glpK* | *cyoD, cyoC,* STM1253*, ydgQ, nifU, rfaB, atpF, ppa* |
| Carbohydrate transport and metabolism | 12 | 4.3 | *gapA,* STM2758*, glgC, yiaN, yihS, malE* | *ptsH, crr, talA, yraO, ptsN, ptsO* |
| Amino acid transport and metabolism | 15 | 5.3 | STM3082*, dppA, glnA, aspA, yjfC* | *brnQ, ycaM, potC, yaeS,* STM1634*,* STM1635*,* STM1636*, cysM, gcvH, metF* |
| Nucleotide transport and metabolism | 6 | 2.1 | *udp, purH* | *cmk, purU, nrdE, thiG* |
| Coenzyme transport and metabolism | 9 | 3.2 | *thiC, citX* | *hemB, ribH, ispA, bioD, ribE,* STM4161*, thiE* |
| Lipid transport and metabolism | 2 | 0.7 | *-* | *fabZ, fabI* |
| Inorganic ion transport and metabolism | 12 | 4.3 | *iroN, sitC, nirC* | *apaG, dps, yccK, mdoG, bfr, bfd, yheL, feoA, yibN* |
| Secondary metabolites biosynthesis, transport, and catabolism | 6 | 2.1 | *-* | *entD, acpP,* STM1366*, nhoA, yrbC,* STM4506 |
| General function prediction only | 13 | 4.6 | *yrdA, yieO,* STM4307*, dcuA,* STM4434 | *ycbL, wraB, sscB, adhP, hns,* STM1849*, yqfA, hfq* |
| Function unknown | 14 | 5.0 | *rtcB* | *yabB, ybgF, yljA, ycfD,* STM1586*, yebC, yeeX, yfbU, yfgL, ygaU, yhbC, yhbN, yiiU* |
| Not in COGs | 77 | 27.3 | *traW*, *rna*, STM1485, STM1868A, STM2745, STM2782, STM3791, STM4098 | STM0081, STM0082, STM0307, *hha*, *pagP*, *ybfA*, *ompX*, STM1055, STM1089, *yccJ,* STM1188*, ycfM, pagD,* STM1249*,* STM1254*,* STM1276*, mipA*, *lpp*, *ssaB, ssaD, ssaE, sseB, sseE, ssaG, ssaH, ssaI, ssaK, ssaM, ssaO, ydgT, ynfB,* STM1583, *ugtL*, *yncJ*, STM1629, STM1630, *ycjE,* STM1839, STM1874, STM1941, STM2138, STM2139, STM2225, STM2234, STM2235, STM2245, *ais*, STM2303, *pagK*, STM2655, STM2693, STM2705, STM2747, *csrB,* STM3034*, yggN, yqjB, yqjC, yqjD, yqjE, yqjK, damX,* STM3580*,* STM3916*,* STM4002, STM4317, *cybC,* STM4504, STM4505 |
| Total | 282 | 100 |  |  |
