# Supplementary material for: Coordinated Regulation of Virulence during Systemic Infection of Salmonella enterica Serovar Typhimurium
Source: PLoS Pathog. 2009 Feb 20;5(2):e1000306. doi: 10.1371/journal.ppat.1000306 (PMC2639726; doi:10.1371/journal.ppat.1000306)
Supplement: Table S3 — Primers used in plasmids construction (A) and qRT-PCR (B). (0.06 MB DOC) [file ppat.1000306.s006.doc]

**Table S3.** Primers used in plasmids construction (A) and qRT-PCR (B).

A

| **Name** | **Sequence** | **Used in** |
| --- | --- | --- |
| lacZ-F1 | 5’- AAAGTCGACGAAGTTCCTATTCTCTAGAAAG -3’ | pFssaGTC |
| lacZ-R1 | 5’- TTGCTCGAGGCCGCGATTAAATTC -3’ | pFssaGTC |
| slyA-F1 | 5’- CCCGAATTCATAACTTAGCAAGCTAATTATAAG -3’ | pBAD30SlyA |
| slyA-R1 | 5’- AAATCTAGATGAATAAAACCCAGGGTGTGGAAC -3’ | pBAD30SlyA |
| spvR-F1 | 5’- TTTTGGATCCGGTTGGTGATTTGTGATGTTTGG -3’ | pWSK29spvR |
| spvR-R1 | 5’- TTTTGAATTCCATGCTCCGCGGTGAACTACCG -3’ | pWSK29spvR |
| ssaG-CLF1 | 5’- AATAGTCGACGTCTTATCTACCATCATAAACATC -3’ | pFssaGTC |
| ssaG-CLR1 | 5’- GTGCGTCGACGATAATGCTTTTCCTTAAAATAAAT -3’ | pFssaGTC |
| ssrB-F1 | 5’- AGCGAATTCAGGTTAATTTTCGCGAGGGCAGC -3’ | pBAD30SsrB/pBAD33SsrB |
| ssrB-R1 | 5’- CTCGAATTCAGGTAGAATACGACATGGTAAAGC -3’ | pBAD30SsrB/pBAD33SsrB |

B

| **Gene** | **Forward primer (5' to 3')** | **Reverse primer (5' to 3')** |
| --- | --- | --- |
| *ssrB* | CCGCAGGTGCTAATGGCTAT | TTGGGTCAATGTAACGCTTGTT |
| *ssaE* | CCGCAGCAATATCAGCAAAA | AAGTGCGCTGTTATGGTAACGA |
| *sseA* | AAAGGCTGCGTTTAGTGAATATCG | TGACTCACCTTAGCCCGGATT |
| *sscA* | GGCTCGCTGCGTATGTTGTT | GCCGGCGAATTCTTTTACCT |
| *ssaG* | ATGATTTGCTCAACCCAGAA | TTTAGCAATGATTCCACTAAGCA |
| *ssaH* | TTCCCAGGTACATGCGATGTTA | TCATTTAAACCCGCCAACAATA |
| *ssaN* | GATGCAACGTCTGAGGCTGAA | GGCAACCACGCATTTAACAA |
| *spvR* | ACTCTTATCCCAACCGAATTTGC | ACCCGTAGGTCCGATTTCCT |
| *fruR* | GTGATCCCGGACCTTGAAAA | AGAACAGGCGATCAGCAGTTG |
| *himD* | GGTTGAAGACGCGGTAAAAGAG | TGCAAAGAAAAACTGCCGAAA |
| *phoP* | TCCGGATATCGCTATTGTCGAT | ACCAGAACCGGCAGTGAAAC |
| *slyA* | TACGCACGTTGGATCAACTTG | CCGCTTTTTCGGTCAGTTTAA |
| *hnr* | TTTCGCTCGCTTCTGGATTC | GGTAAAACGCCCCATCAACTC |
| *rpoE* | ATTGAGTCCCTCCCGGAAGA | CACCGGACAATCCATGATAGC |
| *smpB* | GTCGCCTCGACACATGTTGT | TTCACGATTAATACGGCCATACA |
| *csrA* | AGGTGCGTATTGGCGTGAAC | AGTAACTGGACTGCTGGGATTTTT |
| *rpoS* | CAGCCGTATGCTTCGTCTCA | TTTTCATCGGCCAGGATGTC |
| *crp* | CCAGGTCAACCCGGATATTCT | ACGTCAAGGAAGGCGAGGTTA |
| *ompR* | CGTGAATCTTTCCATCTCATGGT | TCGGCATTGGATTACTTTGACTAC |
| *hfq* | TGGTGAATGGTATTAAGCTGCAA | TCGCGTGCTTATAAACCATCTG |
| *gyrB* | GGCTACAGCAAAAAAGCCAAA | GGAGAATTTCGGATCCGGTACT |
| *rpoD* | GCATCTGGGTGATTTCATCGA | CCAGCCAAAACGTCGTGAGT |
